# Molecular Epidemiology and Antimicrobial Resistance of *Klebsiella pneumoniae* Strains Isolated From Dairy Cows in Xinjiang, China

**DOI:** 10.1002/vms3.70120

**Published:** 2024-11-25

**Authors:** Kuojun Cai, Min Xu, Lu Liu, Hongqiong Zhao

**Affiliations:** ^1^ College of Veterinary Medicine Xinjiang Agricultural University Urumqi Xinjiang China; ^2^ Urumqi Animal Disease Control and Diagnosis Center Urumqi Xinjiang China; ^3^ Urumqi Dairy Association Urumqi Xinjiang China

**Keywords:** antimicrobial resistance, *Klebsiella pneumoniae*, mastitis, molecular epidemiology, raw milk

## Abstract

**Background:**

*Klebsiella pneumoniae* is an opportunistic pathogen that causes severe infections in humans and animals. Nonetheless, little is known about the molecular epidemiology of mastitis‐associated *K. pneumoniae* in dairy cows.

**Objectives:**

This cross‐sectional study investigated the epidemiology and antimicrobial resistance (AMR) of *K. pneumoniae* in 700 milk samples collected from cows with and without mastitis in seven dairy farms in Xinjiang, China.

**Methods:**

*K. pneumoniae* was identified by PCR amplification of the *khe* gene and the automated VITEK 2 Compact System. Resistance against 18 antimicrobial agents was analysed by broth microdilution. Forty‐four new strains were sequenced by whole‐genome sequencing (WGS). WGS data were searched for the presence of AMR and virulence genes. Genotypic characterization was performed by multilocus sequence typing and the analysis of *wzi* allele types and K and O antigens.

**Results:**

*K. pneumoniae* isolates were found in 131 samples (18.7%). The prevalence of *K. pneumoniae* in cows with clinical and subclinical mastitis was higher than that in healthy cows (27.1%, 23.2% and 7.3%, respectively). WGS identified 27 *wzi* allele types, 16 K antigen serotypes, 6 O antigen serotypes and 25 sequence types. Phylogenetic analysis showed high genomic diversity in *K. pneumoniae*. The rate of resistance to tetracycline and cefazolin was 39.7% and 31.3%, and the multidrug resistance rate was 26.7%. Thirty‐nine AMR genes conferring resistance to nine antibiotic classes and 57 virulence genes were identified in new isolates. AMR and virulence genes were more prevalent in known human isolates than in new isolates.

**Conclusions:**

These results improve our understanding of the epidemiology and resistance status of mastitis‐associated *K. pneumoniae* strains. The emergence and spread of multidrug‐resistant *K. pneumoniae* strains threaten food safety and public health.

## Graphical abstract

WGS identified 27 *wzi* allele types (2 new), 16 K antigen serotypes, 6 O antigen serotypes, 25 sequence types (3 new), 39 antibiotic resistance and 57 virulence genes in *Klebsiella pneumoniae* from milk samples of cows with and without mastitis. Phylogenetic analysis showed high genomic diversity in *K. pneumoniae*. Multidrug resistance and hypervirulent rates of the isolates were 26.71% and 19.85%.

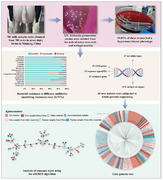

## 1 Introduction

*Klebsiella pneumoniae* is a bacterial pathogen that causes zoonotic infections, including mastitis and infections in the respiratory and urinary tract, soft tissues and bloodstream (Massé, Dufour, and Archambault 2020). The high virulence and antimicrobial resistance (AMR) of *K. pneumoniae* limit the effectiveness of antibiotic therapies (Tsuka et al. 2022; Paulin‐Curlee et al. 2008; Osman et al. 2014; Yang et al. 2019; Cheng et al. 2021). Thus, *K. pneumoniae* is a critical priority for researching and developing new antibiotics (World Health Organization 2017).

Mastitis is a common infectious disease that affects the oestrus and pregnancy of dairy cows and causes high economic losses in the dairy industry by decreasing milk production and quality (Käppeli et al. 2019). Mastitis threatens human and animal health by transferring antimicrobial‐resistant bacteria and causing food poisoning (Li, Chen, and Chen 2019). Among mastitis‐associated bacterial pathogens, *K. pneumoniae* causes the largest loss in milk production (Schukken et al. 2012).

Antibiotic misuse and overuse have become a global public health threat by increasing the prevalence of multidrug‐resistant (MDR) strains (Osman et al. 2014; Yang et al. 2019). Several outbreaks of MDR *K. pneumoniae* have been reported (Arteaga‐Livias et al. 2022). The spread of virulence factors through horizontal gene transfer promotes the emergence of new MDR hypervirulent *K. pneumoniae* strains. In addition, the spread of extended‐spectrum β‐lactamase (ESBL)‐producing strains limits the effectiveness of antimicrobial therapies (Chong, Shimoda, and Shimono 2018). Many mobile AMR genes have been found in *K. pneumoniae* (Navon‐Venezia, Kondratyeva, and Carattoli 2017). *K. pneumoniae* transports these genes between the environment, humans, animals and clinically important bacteria (Wyres and Holt 2018). Furthermore, the AMR and virulence of *K. pneumoniae* in cows vary across geographical regions (Zheng et al. 2022; De Jong et al. 2018; Yang et al. 2019; Gao et al. 2019).

Siderophore systems and capsular K1, K2 and K54 serotypes are associated with hypervirulence (Holden et al. 2016; Wyres et al. 2016). In addition, gene clusters encoding type 1 and 3 fimbriae, allantoinase, iron acquisition systems (Tsuka et al. 2022; Paulin‐Curlee et al. 2008; Osman et al. 2014) and the type VI secretion system (T6SS) are strongly implicated in *K. pneumoniae* pathogenesis in humans and animals (Paczosa and Mecsas 2016). Nonetheless, the presence of these genes in mastitis‐causing *K. pneumoniae* is unknown.

Several *K. pneumoniae* strains that cause bovine mastitis have been identified. Nonetheless, little is known about host preferences and the epidemiological characteristics of *K. pneumoniae*. This cross‐sectional study evaluated the prevalence, AMR and virulence genes of *K. pneumoniae* strains isolated from dairy cows in Xinjiang, China. In addition, an epidemiological analysis was performed to determine genetic diversity, reservoirs and transmission routes.

## 2 Materials and Methods

### 2.1 Farms and Sample Collection

Samples were collected from seven commercial dairy farms (designated A to G) in Xinjiang, China, from January 2021 to December 2022. The number of lactating cows in farms A, B, C, D, E, F and G was 700, 1250, 3200, 2600, 270, 330 and 820, respectively. The number of cows raised in farms E and F was relatively small, and the sanitary conditions were relatively poor. Lactating cows were milked three times per day in milking parlours. Clinical mastitis (CM) symptoms include elevated body temperature, redness, swelling and pain in the udder, as well as the presence of clots in milk. Three forms of CM have been recognized based on clinical signs: mild (abnormal milk), moderate (abnormal milk and udder) and severe (abnormal milk, udder and cow) (Pinzón‐Sánchez, Cabrera, and Ruegg 2011). According to the People's Republic of China Agricultural Industry Standard NY/T2692‐2015, subclinical mastitis (SCM) was defined as > 500,000 somatic cells per mL of milk sample and no visible pathological changes. Animal care was provided by a veterinarian, and several antibacterial agents were used for prophylaxis and treatment. The first, second and third choices of antibiotics were cefquinome sulphate (intramammary infusion), ceftiofur sodium (injection) and cefazolin sodium (injection). Other antibiotics used were amoxycillin sodium/clavulanate potassium, lincomycin hydrochloride (intramammary infusion), florfenicol (injection), tetracycline hydrochloride (injection), compound sulphamethoxazole (injection), kanamycin sulphate (injection) and gentamicin sulphate (gentamicin sulphate injection). The average number of antibiotics was five based on product user manuals.

The udders and teats of cows were cleaned, dried and disinfected with 70% ethanol before sample collection. The first three streams of milk were discarded. Then, milk samples were collected from the four quarters of healthy cows or all infected quarters of mastitis cows using aseptic techniques and combined. The samples were stored at 4°C and transported to the laboratory for bacterial culturing and identification within 24 h of collection.

### 2.2 Isolation and Identification of *K. pneumoniae*

Each of the 700 samples was inoculated in 5 mL of trypticase soy broth (Haibo Biotech, Qingdao, China) and incubated at 37°C for 18–24 h. Then, a loopful of broth culture was streaked onto MacConkey‐inositol‐adonitol‐carbenicillin (MIAC) agar (Haibo Biotech, Qingdao, China) and incubated at 37°C for 18–24 h. A single pink mucoid and nonhemolytic colony was picked and purified on MIAC agar. Gram staining was performed using a Gram staining kit (Baso, Zhuhai, China). The genomic DNA of purified bacteria was extracted using the boiling method (Kuang 2015). Isolates were identified by polymerase chain reaction (PCR) amplification of the *khe* gene and using the automated VITEK 2 Compact System (bioMerieux, France) (Wu et al. 2021). The following primers were used: *khe*‐F 5′‐ATGAAACGACCTGATTGCATTCGC‐3′, *khe*‐R 5′‐TTACTTTTTCCGCG GCTTACCGTC‐3′. PCR products were checked by agarose gel electrophoresis and sequenced (Novogene Company, Beijing, China). Sequences were searched against the GenBank database using the Basic Local Alignment Search Tool (BLAST).

### 2.3 String Test

Hypermucoviscosity (hypervirulence) was assessed using the string test and was defined by the presence of a mucoviscous string longer than 5 mm upon stretching the colony on the agar plate (Fang et al. 2004).

### 2.4 Whole‐genome Sequencing and Assembly

Forty‐four new isolates were selected for whole‐genome sequencing (WGS). Genomic DNA was extracted using the HiPure Bacterial DNA Kit (Magen, Guangzhou, China). WGS was performed on an Illumina NovaSeq 6000 platform (Novogene Company, Beijing, China). Sequencing reads were assembled using SPAdes. Sequence quality control was assessed using FastQC and MultiQC. Raw sequence data were submitted to NCBI GenBank under the accession numbers listed in Table S1.

### 2.5 Bioinformatic Analysis

WGS data were searched against the NCBI database to identify *K. pneumoniae*. Multilocus sequence typing (MLST) was performed using the Pasteur Institute MLST database (www.bigsdb.pasteur.fr) (Diancourt et al. 2005). Capsular locus and lipopolysaccharide (LPS) O antigen types were identified using Kaptive (https://github.com/katholt/Kaptive) (Wyres et al. 2016). The assembled genome was blasted against ResFinder and the Virulence Factor Database (VFDB) to identify AMR and virulence genes (Chen et al. 2016; Zankari et al. 2012) using the following parameters: nucleotide identity, 90%; length coverage, 90%. Moreover, 95 publicly available *K. pneumoniae* sequences (48 from humans, 22 from cattle, 18 from other animals and 7 from the environment) were included in the analysis (Table S2).

### 2.6 Phylogenetic Analysis

The core genome tree of *K. pneumoniae* was constructed using Pathogenwatch (https://pathogen.watch/) (Argimón et al. 2021). The tree was based on genetic distances and assignment to the closest reference genome in a taxonomically representative set. The split network was edited online using iTOL based on a core genome tree. Sequence types (STs) were assigned to each *K. pneumoniae* genome according to seven highly conserved MLST genes (*gapA*, *infB*, *mdh*, *pgi*, *phoE*, *rpoB* and *tonB*) retrieved from the Institut Pasteur MLST database (https://bigsdb.pasteur.fr/). Evolutionary relationships were analyzed using the Based Upon Related Sequence Types (BURST) algorithm and visualized using PHYLOViZ (https://online.phyloviz.net/index) (Francisco et al. 2012).

### 2.7 Antimicrobial Susceptibility Testing

The susceptibility of *K. pneumoniae* isolates to 18 clinically significant broad‐spectrum antimicrobial agents commonly used on dairy farms was determined using the broth microdilution method recommended by the Clinical and Laboratory Standards Institute (CLSI) 2021. The agents included cephalosporins (cefazolin, cefotaxime, ceftazidime, cefepime, cefoxitin, ceftiofur), β‐lactam/β‐lactamase inhibitors (cefotaxime/clavulanic acid, ceftazidime/clavulanic acid), carbapenems (meropenem), chloramphenicol (chloramphenicol, florfenicol), sulphonamides (sulphamethoxazole), aminoglycosides (gentamicin, streptomycin), tetracyclines (tetracycline, glycine tetracycline [tegacyclin]), quinolones (ciprofloxacin) and polypeptides (polymyxin E). The results were interpreted according to the CLSI‐M100‐Ed31 and VET01S‐Ed5 standards (2020, 2021). The interpretation of MICs for streptomycin and tegacyclin was based on the European Committee on Antimicrobial Susceptibility Testing breakpoints.

Resistance phenotypes were classified as MDR, extensively drug‐resistant and pandrug‐resistant, as previously described (Magiorakos et al. 2012). A susceptible strain (*Escherichia coli* ATCC 25922) and a *bla*_SHV_‐positive strain (*K. pneumoniae* ATCC 700603) were included to monitor the performance of ESBL detection agents and as quality controls for the susceptibility tests.

### 2.8 Statistical Examination

Chi‐square tests were performed using SPSS version 26.0, and a *p* value of less than 0.05 was considered statistically significant. All tests were two‐tailed.

## 3 Results

### 3.1 Prevalence of *K. pneumoniae* Across Dairy Farms

*K. pneumoniae* was identified in 131 raw milk samples (18.7%) by PCR amplification of *khe* and biochemical analysis (Figure S1A). The prevalence of *K. pneumoniae* in farms A, B, C, D and G was 17.0% (17/100), 18.0% (18/100), 15.3% (23/150), 14.7% (22/150) and 17.0% (17/100) (Table 1). Prevalence was significantly higher in farms F (36.0%, 18/50) (*χ* ^2^ = 12.2, *p *< 0.05) and E (32.0%, 16/50) (*χ* ^2^ = 8.1, *p *< 0.05) than in the other dairy farms (average, 16.2%; 97/600). Moreover, the prevalence was significantly higher in cows with CM and SCM (27.1% [26/96], *χ* ^2^ = 22.8, *p *< 0.05; 23.2% [89/384], *χ* ^2^ = 24.6, *p *< 0.05) than in healthy cows (7.3%, 16/220).

**TABLE 1:** Prevalence of *Klebsiella pneumoniae* strains isolated from lactating cows in seven dairy farms in Xinjiang, China.

Dairy farms	A (*n* = 700)			B (*n* = 1250)			C (*n *= 3800)			D (*n *= 2600)			E (*n *= 270)			F (*n *= 330)			G (*n* = 820)			
Quality of raw milk	H	SCM	CM	H	SCM	CM	H	SCM	CM	H	SCM	CM	H	SCM	CM	H	SCM	CM	H	SCM	CM	Total
Number of milk samples	40	50	10	30	55	15	40	80	30	40	90	20	20	25	5	20	24	6	30	60	10	700
Number of isolates	2	12	3	2	11	5	1	16	6	2	16	4	3	10	3	4	11	3	2	13	2	131
Rate of detection (%)	5.0	24.0	30.0	6.7	20.0	33.3	2.5	20.0	20.0	5.0	17.8	20.0	15.0	40.0	60.0	20.0	45.8	50.0	6.7	21.7	20.0	18.7

*Note*: H, SCM and CM correspond to milk samples collected from healthy cows, cows with SCM and cows with clinical mastitis, respectively.

### 3.2 String Test

Of 131 isolates grown on agar plates, 26 (19.9%) had a hypermucoviscous (hypervirulent) phenotype (Figure S1B). However, the regulator of mucoid phenotype gene *rmpA* was not detected in new strains.

### 3.3 Whole‐Genome Structure of *K. pneumoniae* Populations

**FIGURE 1:**
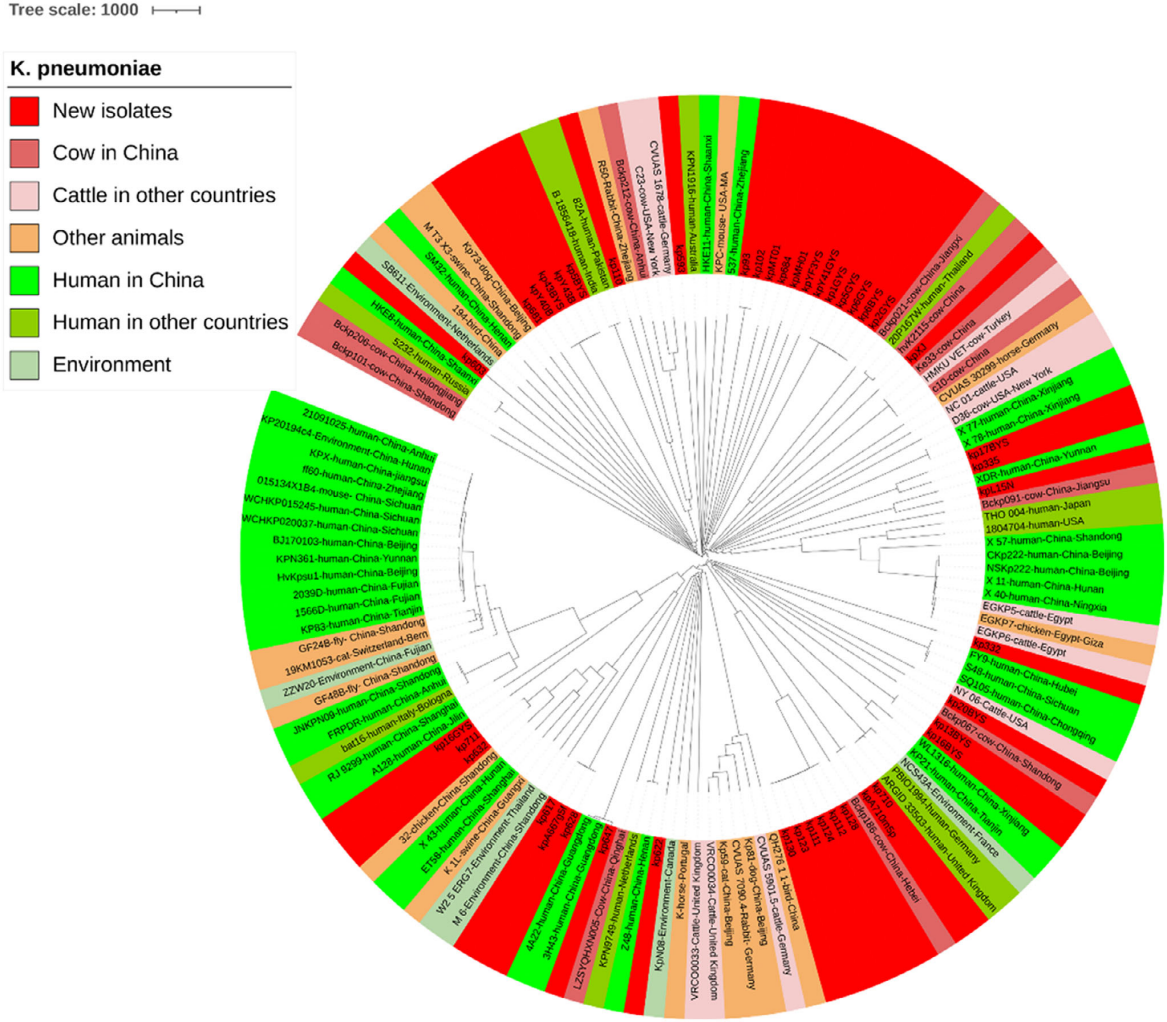
Core genome tree of 44 newly identified *Klebsiella pneumoniae* strains isolated from lactating cows in seven dairy farms in Xinjiang, China, and 95 publicly available genomes of *K. pneumoniae* strains isolated from humans (48), cattle (22), other animals (18) and the environment (7).

To locate *K. pneumoniae* strains in the phylogenetic tree, we constructed a core genome tree based on 139 whole‐genome sequences, including 44 new sequences and 95 publicly available sequences (Figure 1). The results showed high genetic diversity. The core genome tree comprised two subgroups, with 22 strains in subgroup 1 and 117 in subgroup 2, indicating the high prevalence of *K. pneumoniae* in the latter. Newly identified sequences were highly homologous to database sequences based on the core genome.

**FIGURE 2:**
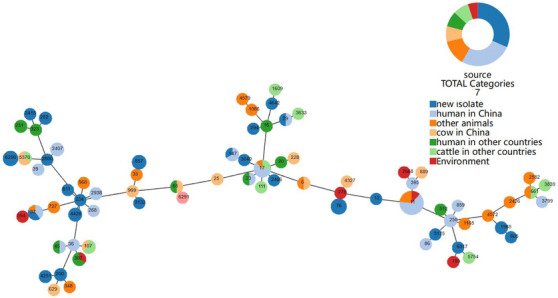
BURST map of sequence types (STs) detected in 139 *Klebsiella pneumoniae* strains. The map was based on seven highly conserved housekeeping genes from the BIGSdb database and was visualized using PHYLOViZ. Each circle represents one ST, and the size indicates the number of strains. Branch lengths indicate the distance of each ST. The colours indicate the percentages of STs in different sources.

ST distributions indicated high genetic diversity in mastitis‐associated *K. pneumoniae* strains (Figure 2). Seventy‐five STs were identified in 139 whole‐genome sequences. The most prevalent types were ST11 (12.2%, 17/139), ST17 (5.8%, 8/139), ST76 (4.3%, 6/139) and ST6290 (3.6%, 5/139). Strains with ST11 were more predominant in China (94.1%, 16/17) and human samples (64.7%, 11/17). In addition, ST37 (2.9%, 4/139), ST147 (2.2%, 3/139) and ST29 (1.4%, 2/139) were both detected in new isolates and human isolates from China.

### 3.4 Capsular Serotyping and MLST

Twenty‐seven *wzi* alleles were identified in 44 new sequences (Table S3), and the most prevalent were wzi100‐KL10 (13.6%, 6/44), wzi442‐KL167 (13.6%, 6/44) and wzi181‐KL133 (9.1%, 4/44); wzi737‐KL48 (*n* = 1) and wzi738‐KL18 (*n* = 1) were newly identified. Sixteen K types were identified. The most common K type was K10 (13.6%, 6/44), and serotypes K1 (*n* = 1) and K54 (*n* = 1) from hypervirulent clones were found. In addition, six LPS O antigen serotypes were identified: O3b (27.3%, 12/44), O3/O3a (18.2%, 8/44), OL101 (18.2%, 8/44), O2a (15.9%, 7/44), O1 (15.9%, 7/44) and O4 (4.6%, 2/44) (Table S3).

Twenty‐five STs were identified, and the most prevalent were ST76 (13.6%, 6/44), ST6290 (11.4%, 5/44) and ST857 (9.1%, 4/44) (Table S3). Three new MLST genotypes (ST6290 [2‐1‐5‐1‐604‐8‐13], ST6291 [51‐1‐5‐1‐9‐357‐13] and ST6317 [3‐16‐1‐1‐20‐1‐40]) were identified and were added to the *K. pneumoniae* MLST database. ST11 and ST23, previously identified in liver abscess patients and pneumonia patients, respectively, were not detected in our samples. The phylogenetic tree showed the presence of several serotypes and genotypes in new isolates. Strains of the same serotype may have different genotypes, and strains of the same genotype may have different serotypes.

### 3.5 Prevalence of Virulence Genes

**FIGURE 3:**
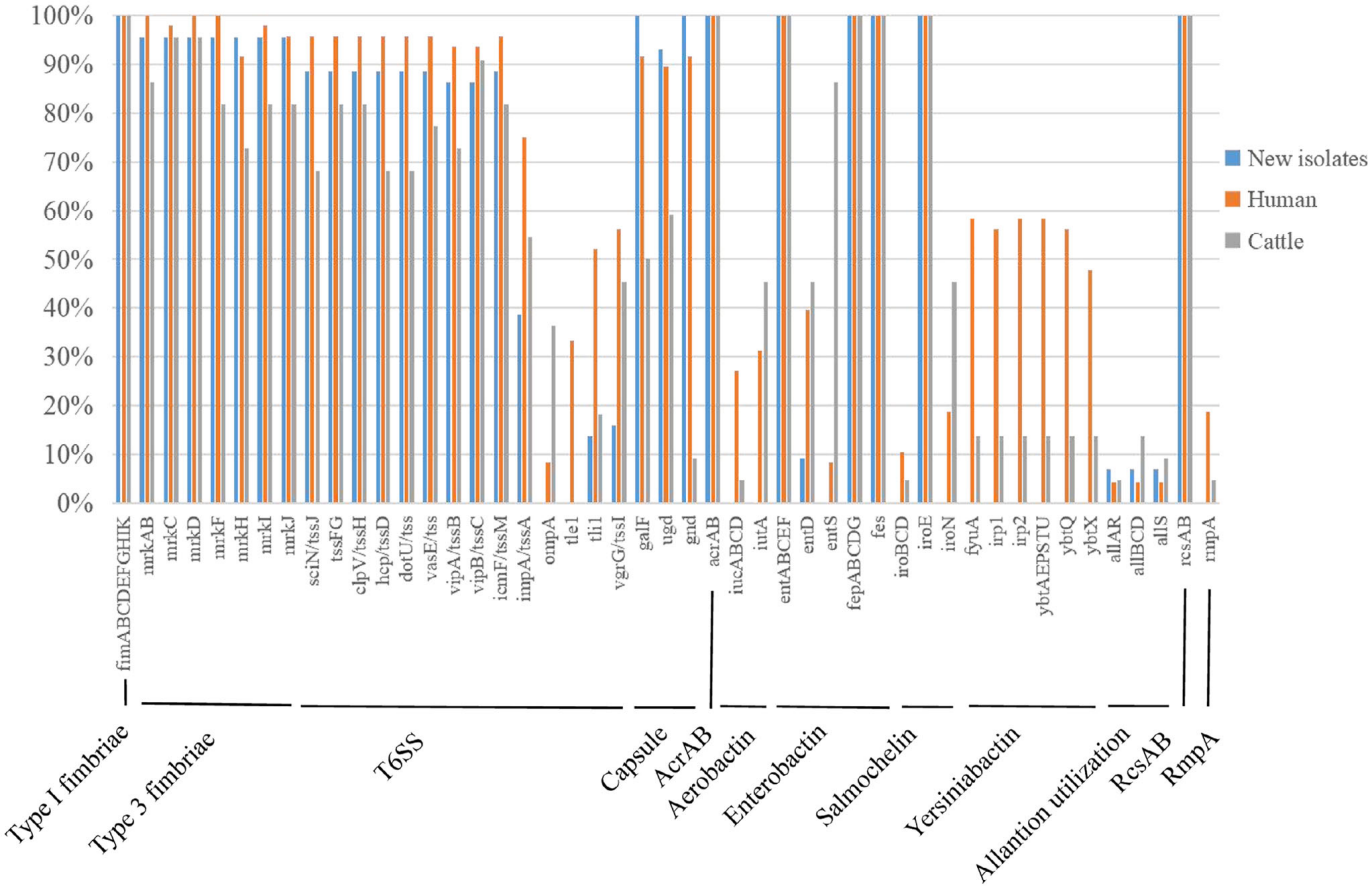
Major virulence genes in the genomes of 44 *Klebsiella pneumonia* strains isolated from lactating cows in seven dairy farms in Xinjiang, China, and in known human and cattle isolates.

One hundred fourteen genomes from 44 newly identified strains, 48 human isolates and 22 cattle isolates were assessed for the presence of virulence genes (Figure 3). Fifty‐seven important virulence genes encoding nine virulence factors were detected in new isolates (Figure 3, Table S3). Virulence genes encoding type I fimbriae (*fimA*‐*I*, *fimK*), enterobactin (*entA*‐*F*, *fepA*‐*G*, *fes*), regulators of capsular polysaccharide synthesis (*rcsA* and *rcsB*), efflux pumps (*acrA* and *acrB*) and salmochelin (*iroE*) were identified in the three groups. The prevalence of *entD* in human, cattle and new strains was 39.6%, 45.5% and 9.1%, respectively, and the detection rate of *entS* in human and cattle isolates was 8.3% and 86.4%, respectively; *entS* was not detected in the new isolates. The prevalence of type 3 fimbriae genes (*mrkA*‐*D*, *mrkF*, *mrkH*‐*J*) in the three groups was 72.7%–100%.

The prevalence of T6SS genes (*sciN*/*tssJ*, *tssF*‐*G*, *clpV*/*tssH*, *hcp*/*tssD*, *dotU*/*tss*, *vasE*/*tss*, *vipA*/*tssB*, *vipB*/*tssC*, *icmF*/*tssM*) in newly sequenced human and cattle isolates was 86.4%–88.6%, 93.8%–95.8% and 68.2%–90.9%, respectively. However, the frequency of other T6SS genes (*impA*/*tssA*, *ompA*, *tle1*, *tli1*, *vgrG*/*tssI*) was much lower in new strains (38.6%).

The prevalence of yersiniabactin genes (*fyuA*, *irp1*, *irp2*, *ybtA*, *ybtE*, *ybtP*, *ybtQ*, *ybtS*, *ybtT*, *ybtU*, *ybtX*) in human and cattle isolates was 47.9%–58.3% and 13.6%. Aerobactin genes (*iucA*‐*D*, *iutA*), salmochelin genes (*iroB*‐*D*, *iroN*) and *rmpA* were not detected in new isolates. Capsule genes (*galF*, *ugd*, *gnd*) were significantly more predominant in new strains (93.2%–100%) and human isolates (89.6%–91.7%) than in cattle isolates (9.1%–59.1%). The overall frequency of allantoin genes (*allA*‐*D*, *allR*, *allS*) was 4.2%–13.6%.

### 3.6 Antimicrobial Susceptibility Testing

**FIGURE 4:**
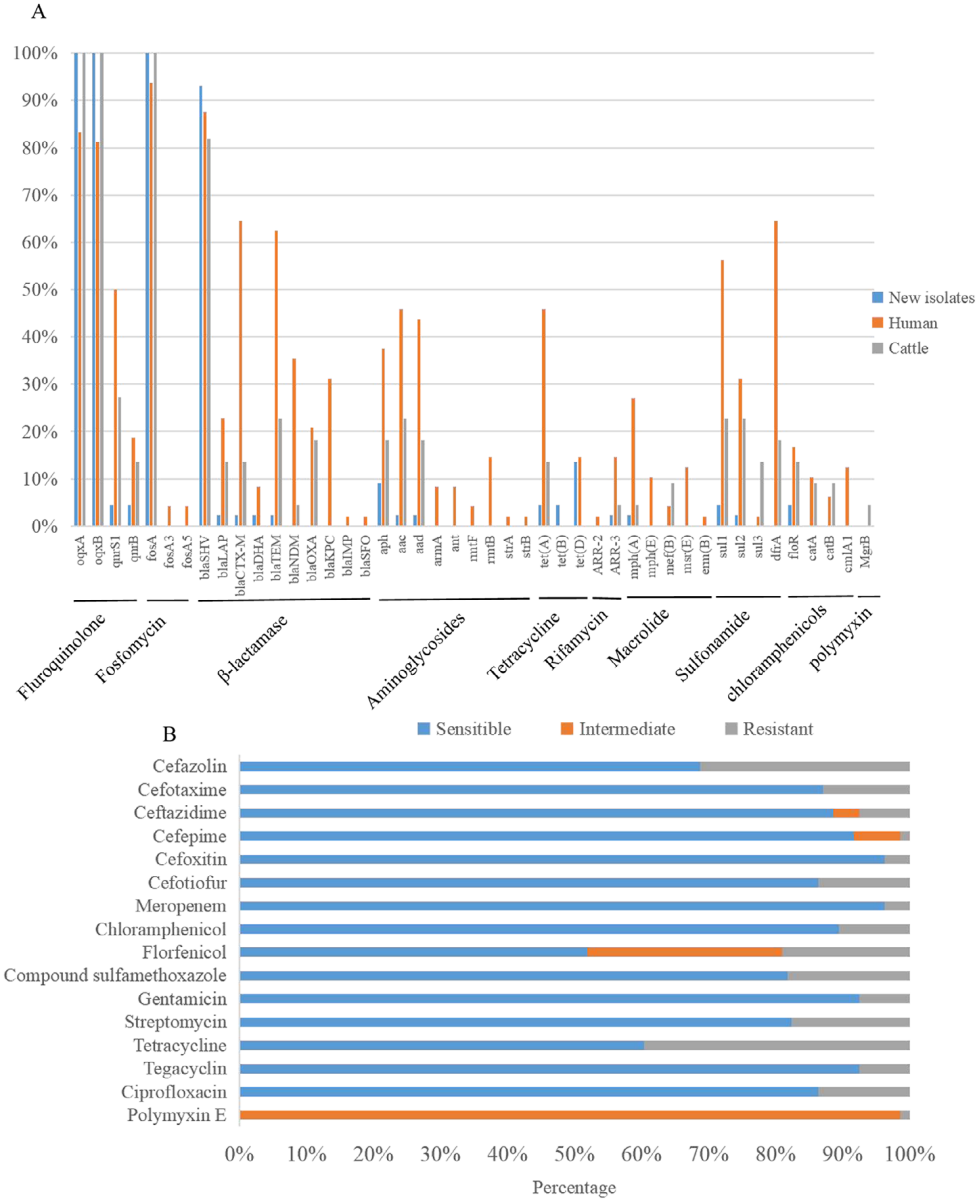
Resistance genes and phenotypes. (A) Antimicrobial resistance genes in the genome of 44 newly identified *Klebsiella pneumoniae* strains isolated from lactating cows in seven dairy farms in Xinjiang, China, and in known human and cattle isolates. (B) Rate of resistance of 131 *K. pneumoniae* isolates to 16 antibiotics.

The susceptibility test results are shown in Figure 4B. The rate of resistance to meropenem and cefoxitin was 3.8%, and the rate of resistance to tetracycline and cefazolin was 39.7% and 31.3%. The rate of resistance to cefotaxime, ceftiofur, chloramphenicol, florfenicol, sulphamethoxazole and streptomycin varied from 10.0% to 20.0%, and the rate of resistance to ceftazidime, cefepime, gentamicin, tegacyclin, ciprofloxacin and polymyxin E was less than 10.0%. In addition, 26.7% (35/131) of strains were MDR, and none were extensively drug‐resistant or pandrug‐resistant. Twenty‐six strains (19.9%) were ESBL producers.

### 3.7 Prevalence of AMR Genes

Thirty‐nine genes conferring resistance to nine antibiotic classes (fluoroquinolone, fosfomycin, β‐lactam, aminoglycoside, tetracycline, rifamycin, macrolide, sulphonamide and chloramphenicol) were detected in new isolates (Figure 4A; Table S3). Thirty‐four strains carried four AMR genes (77.3%), and one strain carried 18 AMR genes. β‐Lactam resistance genes were found in 95.5% of the isolates. Forty strains (95.3%) carried one β‐lactam resistance gene, and two strains carried two and three β‐lactam resistance genes, respectively. The most prevalent β‐lactam AMR gene was *SHV* (97.6%, 41/42); 14 *bla*_SHV_ types were found, and *bla*_SHV‐40_ (17.1%) was the most prevalent. Quinolone resistance genes *oqxA* and *oqxB* and the fosfomycin resistance gene *fosA* were found in all strains. *Tet* was detected in 10 isolates (22.7%); of these, 60.0%, 20.0% and 20.0% carried *tet*(D), *tet*(A) and *tet*(B), respectively.

The presence of AMR genes was assessed in newly sequenced human and cattle isolates. The genes *oqxA*, *oqxB* and *fosA* were identified in all strains, and *bla*_SHV_ was present in most strains. Sixteen AMR genes were found exclusively in human isolates. The prevalence of genes *bla_CTX_*_‐_ *_M_*, *aph*, *aac*, *aad*, *tet*(A), *ARR‐3*, *mph*(A), *sul1* and *dfrA* was higher in human isolates. In addition, 72.3% of new strains and 54.6% of cattle strains carried four AMR genes. The number of AMR genes in human isolates varied from 3 to 24. The average number of AMR genes in newly identified human and cattle isolates was 5.2, 12.5 and 8.1.

## 4 Discussion

*K. pneumoniae* is a widespread pathogen causing mastitis in dairy cows. Although *K. pneumoniae* causes fatal hospital‐ and community‐acquired infections in humans (Marr and Russo 2019), the epidemiology of *K. pneumoniae* associated with bovine mastitis and underlying pathophysiological mechanisms are unclear. This study evaluated the prevalence, AMR, and molecular characteristics of 44 new strains isolated from dairy cows with and without mastitis and previously identified strains.

Environmental opportunistic pathogens commonly cause mastitis in dairy cows (Ruegg 2017). CM caused by *K. pneumoniae* is characterized by a longer subclinical phase, severe inflammation and poor clinical outcomes (Fuenzalida and Ruegg 2019). In dairy farms, *K. pneumoniae* is frequently recovered from the environment, nasal swabs, anal swabs, teat‐end swabs and mastitis milk samples (Rowbotham and Ruegg 2016). The molecular epidemiology and genetic diversity of mastitis‐associated *K. pneumoniae* in Chinese dairy farms are incompletely understood (Cheng et al. 2021; Song et al. 2022).

The detection rate of *K. pneumoniae* in raw milk samples varies by season (Fu et al. 2022; Paulin‐Curlee et al. 2008). Moreover, the prevalence of *K. pneumoniae* in milk samples was 11.9% in Cairo, Egypt (Osman et al. 2014) and 25.7% in Peshawar, Pakistan (Saddam et al. 2023). *K. pneumoniae* was found in 18.7% of our samples. The prevalence was significantly higher in farms F and E (36.0% and 32.0%) than in other farms, which can be attributed to opportunistic infections resulting from faecal contamination and poor sanitary conditions.

*K. pneumoniae* is ubiquitous in the environment (Wareth and Neubauer 2021). The genetic diversity of *K. pneumoniae* is determined by several factors, including nipple cleanliness, infection sources and transmission patterns (Fuenzalida and Ruegg 2019). Therefore, dairy farms should strengthen prevention and treatment methods and implement feed and production management technologies to reduce disease transmission.

Finding the sources of infection and interrupting disease transmission is essential for disease control. Genotyping can help identify the sources of infection and transmission routes. MLST is widely used because of its high throughput, high resolution and reproducibility and easy data sharing (Pérez‐Losada et al. 2013). *K. pneumoniae* STs considered important and highly prevalent globally include ST11, ST15, ST23, ST65, ST86, ST258, ST395, ST494 and ST512 (Argimón et al. 2021). Seventy‐five STs were identified in 139 whole‐genome sequences, including 44 new and 95 publicly available sequences. The most prevalent type was ST11 (12.2%, 17/139), among which 11 strains were isolated from human samples from China (64.7%, 11/17). This result is because ST11 carbapenem‐resistant *K. pneumoniae* has become the dominant clone in China, and such strains may cause severe infections that are difficult to treat with antibiotics (Liao, Liu, and Zhang 2020). Furthermore, ST37, ST147 and ST29 were found in new and human isolates from China, indicating that these ST‐type *K. pneumoniae* strains can infect humans and cows. Core genome phylogeny and MLST revealed high genetic diversity in new *K. pneumoniae* strains. However, more epidemiological research is needed to assess the prevalence of ST types across farms in Xinjiang. STs associated with hypervirulence (ST65 and ST375), multidrug resistance (ST11, ST70 and ST323) and severe blood infections (ST23) were not identified in new strains (Zheng et al. 2022). However, three new STs (*n* = 7) were identified and deposited in the BIGSdb database.

The diversity of ST clones varies across time and location (Argimón et al. 2021), and the high diversity of STs in *K. pneumoniae* strains infecting cows with and without mastitis indicates that no ST clones are highly prevalent in Xinjiang. A core genome tree of *K. pneumoniae* confirmed the high genetic diversity in these strains.

Virulence factors play an essential role in the virulence of *K. pneumoniae* (Wang et al. 2020). Fimbriae, capsule polysaccharides, iron carriers and LPS are involved in the adhesion, invasion and growth of *K. pneumoniae* (Cheng et al. 2020). The capsule contributes to immune evasion mechanisms, including antiphagocytosis, inhibition of early inflammatory responses, neutralization of antimicrobial peptides and inhibition of dendritic cell maturation (Paczosa and Mecsas 2016). Bacteria absorb host iron via siderophores, including aerobactin, salmochelin, enterobactin and yersiniabactin, increasing virulence and the risk of infection (Wang et al. 2020). Type 1 (fim) and type 3 (mrk) fimbriae are commonly found in *K. pneumoniae* (Schroll et al. 2010). Type 1 fimbriae enhance virulence by adhering to mucosal and epithelial surfaces, and type 3 fimbriae adhere to the cell membrane and promote biofilm formation (Schroll et al. 2010). Several virulence factors have been identified in *K. pneumonia*e strains infecting humans (Wang et al. 2020). *AcrA*, *acrB*, *rcsA*, *rcsB*, T6SS genes and genes encoding type 1 and type 3 fimbriae and enterobactin were found in newly sequenced human and cattle isolates. The yersiniabactin gene was significantly more prevalent in human strains (47.9%–58.3%) than in cattle isolates (13.6%) and new strains (0%), consistent with data from the United States (Zheng et al. 2022). Aerobactin and salmochelin genes were not found in new isolates, except *iroE*. The difference in the prevalence of iron‐absorbing molecules yersiniabactin, aerobactin and salmochelin indicates that human strains can produce larger and more active iron‐absorbing molecules compared with bovine strains, which might increase virulence and pathogenicity. The frequency of capsular genes (*galF*, *ugd* and *gnd*) was significantly higher in new isolates (93.2%–100%) and human strains (89.6%–91.7%) than in cattle strains (9.1%–59.1%), indicating that the ability to synthesize capsule polysaccharides varies depending on the bacterial source, and these polysaccharides protect bacteria from phagocytosis and killing by serum factors. Eight newly sequenced strains were string test‐positive. Nonetheless, *rmpA*, which regulates the expression of capsular polysaccharides in hypervirulent strains, was not detected. These virulence genes might contribute to pathogenicity, host adaptation and host specificity (Wang et al. 2020; Paczosa and Mecsas 2016; Schroll et al. 2010). Nonetheless, we found no significant correlation between virulence genes, serotypes and genotypes.

In human outbreaks, *K. pneumoniae* causes severe infections and is commonly resistant to ampicillin, carbapenem, fluoroquinolone, aminoglycosides, colistin or tetracycline (Zheng et al. 2022). Antimicrobial chemotherapy is implemented to prevent and control bovine mastitis. However, with the increasing rates of AMR among mastitis‐associated pathogens infecting dairy cows, antibiotic overuse and misuse might lead to the emergence of pan‐resistant strains (Song et al. 2022). The rates of antibiotic resistance among *K. pneumoniae* isolates from dairy cows vary across regions (De Jong et al. 2018). In parts of Europe and the United States, isolates from CM cases have low levels of resistance to tetracycline (5.6%–19.5%) and β‐lactam antibiotics (0%–6.9%) (De Jong et al. 2018).

The rates of resistance to cefquinome, kanamycin, ceftiofur, polymyxin B and tetracycline among *K. pneumoniae* strains in China are 10.0%–32.0% (Cheng et al. 2019). The rate of resistance to streptomycin, tetracycline and gentamicin in *K. pneumoniae* strains isolated from bovine mastitis in New York State was 29.4%, 5.6% and 4.2% (Yang et al. 2019). The high resistance to tetracycline (39.7%) and cefazolin (31.3%) in our samples might be related to the selective pressure caused by the use of tetracycline and cefazolin in six out of seven dairy farms. Resistance to meropenem in human clinical isolates of *K. pneumonia*e is increasing rapidly in China (Hu et al. 2022). The rate of resistance to ceftiofur (13.7%) in our samples was lower than that in *K. pneumoniae* strains isolated from cows with CM in large Chinese dairy herds in 2019 (21.0%) (Cheng et al. 2019). In contrast, the rate of resistance to tetracycline (39.7%) in our samples was higher than that in strains infecting cows with CM in other countries (Saddam et al. 2023; Nobrega et al. 2021; Massé, Dufour, and Archambault 2020). The MDR rate in our samples (26.7%) was higher than that in Brazil (7.7%) (Nobrega et al. 2021) but lower than that in Pakistan (44.4%) (Saddam et al. 2023), indicating differences in AMR in milk‐derived *K. pneumoniae* isolates across geographical regions.

Few studies have compared AMR genes in *K. pneumoniae* sourced from dairy cows and humans. In our study, cattle isolates carried fewer AMR genes than human strains. The genes *oqxA*, *oqxB* and *fosA* were found in all isolates. Consistent with this finding, these genes were detected in 180 *K. pneumoniae* strains collected from dairy farms in 11 U.S. states (Zheng et al. 2022). The *bla*_SHV_ gene was identified in 93.2% of new isolates, of which *bla*_SHV40_ was the most prevalent (17.1%), indicating that SHV‐type β‐lactamases were the primary cause of resistance of new isolates to β‐lactams, and the *bla*_SHV40_ gene was the most prevalent among 14 identified SHV allelic variants. Thus, accurately identifying these variants is essential for surveillance and analysis of transmission modes. The genes *bla*_KPC_, *bla*_NDM_, *bla*_IMP_ and *bla*_OXA_, associated with MDR in human isolates, were not detected in new isolates, indicating that human isolates carried more carbapenemase genes than cow isolates, causing the rapid spread of carbapenemase‐producing *K. pneumoniae* in humans. Environmental changes and selection pressures might have led to differences in AMR genes among human and cow isolates.

## 5 Conclusions

This study analyzed the epidemiological characteristics, genetic variability and AMR of *K. pneumoniae* strains infecting dairy cows with and without mastitis in Xinjiang, China. These results improved our understanding of the virulence of mastitis‐associated *K. pneumoniae* strains and can help prevent, diagnose and treat mastitis in lactating cows.

## Author Contributions

**Kuojun Cai**: conceptualization (lead), writing–original draft (lead), formal analysis (lead), writing–review and editing (equal). **Min Xu**: writing–review and editing (equal). **Lu Liu**: conceptualization (supporting), writing–original draft (supporting), writing–review and editing (equal). **Hongqiong Zhao**: methodology (lead), review and editing (equal).

## Ethics Statement

All experimental procedures involving animals were approved by the Animal Welfare and Ethics Committee of Xinjiang Agricultural University, Urumqi, Xinjiang, China (Protocol number: 2021081), in accordance with the ethical policies of the journal.

## Conflicts of Interest

The authors declare no conflicts of interest.

## Supporting information



Supporting Information

## Data Availability

The data supporting these findings are available at NCBI, and the accession numbers are listed in Table S1.
