# Supplementary material for: Molecular Epidemiology and Antimicrobial Resistance of Klebsiella pneumoniae Strains Isolated From Dairy Cows in Xinjiang, China
Source: Vet Med Sci. 2024 Nov 25;11(1):e70120. doi: 10.1002/vms3.70120 (PMC11586637; doi:10.1002/vms3.70120)
Supplement: Supplementary file 1 — Supporting Information [file VMS3-11-e70120-s001.docx]

| A |  | B |  |
| --- | --- | --- | --- |
|  | 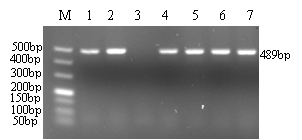 |  | 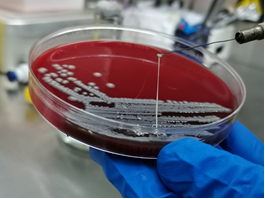 |

**Supplementary Fig. 1.** Identification of *Klebsiella pneumoniae* and String test. A: PCR amplification of the *khe* gene from *Klebsiella pneumoniae* isolates.M: DNA marker 500; 1: positive control; 3: negative control; 2 and 4-7: positive strains. B:String test of *Klebsiella pneumoniae* strains.

**Supplementary Table 1.** List of NCBI genome accessions of all strains in this study.

| **Strain** | **Genome submission** | **BioProject** | **BioSample** |
| --- | --- | --- | --- |
| kp102 | SUB12867896 | PRJNA934766 | SAMN33282845 |
| kp110 | SUB12869340 | PRJNA935033 | SAMN33295593 |
| kp111 | SUB12869404 | PRJNA935038 | SAMN33295795 |
| kp112 | SUB12869434 | PRJNA935042 | SAMN33295929 |
| kp123 | SUB12869438 | PRJNA935044 | SAMN33296000 |
| kp124 | SUB12869440 | PRJNA935046 | SAMN33296017 |
| kp128 | SUB12869483 | PRJNA935055 | SAMN33296045 |
| kp130 | SUB12870073 | PRJNA935074 | SAMN33296277 |
| kp13BYS | SUB12870105 | PRJNA935078 | SAMN33296347 |
| kp16BYS | SUB12870134 | PRJNA935084 | SAMN33296441 |
| kp16GYS | SUB12870144 | PRJNA935085 | SAMN33296472 |
| kp17BYS | SUB12870151 | PRJNA935086 | SAMN33296475 |
| kp1GYS | SUB12870157 | PRJNA935090 | SAMN33297343 |
| kp20BYS | SUB12870179 | PRJNA935095 | SAMN33297791 |
| kp2GYS | SUB12870190 | PRJNA935098 | SAMN33297821 |
| kp332 | SUB12870196 | PRJNA935102 | SAMN33297840 |
| kp335 | SUB12870224 | PRJNA935108 | SAMN33298000 |
| kp43BYS | SUB12870235 | PRJNA935112 | SAMN33298053 |
| kp593 | SUB12870246 | PRJNA935114 | SAMN33298055 |
| kp5BYS | SUB12870258 | PRJNA935115 | SAMN33298057 |
| kp5GYS | SUB12870266 | PRJNA935117 | SAMN33298082 |
| kp603 | SUB12870270 | PRJNA935119 | SAMN33298098 |
| kp617 | SUB12870275 | PRJNA935122 | SAMN33298147 |
| kp622 | SUB12870284 | PRJNA935123 | SAMN33298148 |
| kp628 | SUB12870291 | PRJNA935125 | SAMN33298150 |
| kp632 | SUB12870300 | PRJNA935126 | SAMN33298151 |
| kp647 | SUB12870303 | PRJNA935128 | SAMN33298153 |
| kp681 | SUB12870305 | PRJNA935129 | SAMN33298154 |
| kp684 | SUB12870310 | PRJNA935130 | SAMN33298155 |
| kp6BYS | SUB12870311 | PRJNA935135 | SAMN33298156 |
| kp6GYS | SUB12870313 | PRJNA935133 | SAMN33298157 |
| kp710 | SUB12870319 | PRJNA935137 | SAMN33298159 |
| kp711 | SUB12870328 | PRJNA935138 | SAMN33298160 |
| kp93 | SUB12870333 | PRJNA935140 | SAMN33298161 |
| kpL15N | SUB12870338 | PRJNA935141 | SAMN33298163 |
| kpY40B | SUB12870340 | PRJNA935142 | SAMN33298164 |
| kpY41GYS | SUB12870348 | PRJNA935144 | SAMN33298178 |
| kpY43B | SUB12870351 | PRJNA935145 | SAMN33298179 |
| kpYF3YS | SUB12870361 | PRJNA935146 | SAMN33298180 |
| kpA667g5f | SUB12870367 | PRJNA935149 | SAMN33298182 |
| kpMH01 | SUB12870372 | PRJNA935150 | SAMN33299125 |
| kpXJ | SUB12870378 | PRJNA935151 | SAMN33299126 |
| kpA710m5p | SUB12870382 | PRJNA935153 | SAMN33299127 |
| kpMT01 | SUB12870396 | PRJNA935154 | SAMN33299131 |

**Supplementary Table 2.** Information of 95 publicly available Klebsiella pneumoniae.

| **#** | **Strain** | **Assembly** | **GenBank assembly accession** | **BioSample** | **SRA** | **Source** | **Geographic location** |
| --- | --- | --- | --- | --- | --- | --- | --- |
| 1 | FRPDR | ASM1524323v1 | GCA_015243235.1 |  |  | human | China:Anhui |
| 2 | 21091025 | SAMN27363257 | GCA_022982575.1 |  |  | human | China:Anhui |
| 3 | HvKpsu1 | ASM2429924v1 | GCA_024299245.1 |  |  | human | China:Beijing |
| 4 | BJ170103 | ASM2370062v1 | GCA_023700625.1 |  |  | human | China:Beijing |
| 5 | CKp222 | ASM1168315v1 | GCA_011683155.1 |  |  | human | China:Beijing |
| 6 | NSKp222 | ASM1168318v1 | GCA_011683185.1 |  |  | human | China:Beijing |
| 7 | WCHKP020037 | ASM281130v2 | GCA_002811305.2 |  |  | human | China:Sichuan |
| 8 | WCHKP015245 |  |  | SAMN09842386 | SRS3689838 | human | China:Sichuan |
| 9 | 4A22 | ASM2327302v1 | GCA_023273025.1 |  |  | human | China:Guangdong |
| 10 | 3H43 | ASM2327310v1 | GCA_023273105.1 |  |  | human | China:Guangdong |
| 11 | XDR | ASM78562v1 | GCA_000785625.1 |  |  | human | China:Yunnan |
| 12 | KPN361 | ASM1680402v1 | GCA_016804025.1 | SAMN14752225 | SRS6559796 | human | China:Yunnan |
| 13 | S48 | ASM2029313v1 | GCA_020293135.1 |  |  | human | China:Sichuan |
| 14 | ET58 | ASM2029468v1 | GCA_020294685.1 |  |  | human | China:Shanghai |
| 15 | RJ 9299 | PDT001288606.1 | GCA_023036455.1 | SAMN25166419 | SRS11800523 | human | China:Shanghai |
| 16 | Z48 | ASM2029270v1 | GCA_020292705.1 |  |  | human | China:Zhengzhou |
| 17 | SM32 | ASM342688v1 | GCA_003426885.1 | SAMN09508733 |  | human | China:Zhengzhou |
| 18 | SQ105 | ASM2029280v1 | GCA_020292805.1 |  |  | human | China:Chongqing |
| 19 | FY9 | ASM2443445v1 | GCA_024434455.1 |  |  | human | China:Hubei |
| 20 | X_78 |  |  | SAMN21366071 | SRS10099941 | human | China:Xinjiang |
| 21 | X_77 |  |  | SAMN21366070 | SRS10099940 | human | China:Xinjiang |
| 22 | WL1316 |  |  | SAMN14330249 | SRS6277849 | human | China:Xinjiang |
| 23 | X_40 |  |  | SAMN21366033 | SRS10099903 | human | China:Ningxia |
| 24 | HKE11 |  |  | SAMN08707461 | SRS3058456 | human | China:Shaanxi |
| 25 | HKE8 |  |  | SAMN08707458 | SRS3058445 | human | China:Shaanxi |
| 26 | KP83 |  |  | SAMN18498922 | SRS8590815 | human | China:Tianjin |
| 27 | KP21 |  |  | SAMN18498915 | SRS8590808 | human | China:Tianjin |
| 28 | X_57 |  |  | SAMN21366050 | SRS10099920 | human | China:Shandong |
| 29 | JNKPN09 | PDT001167485.1 | GCA_021900035.1 | SAMN22136649 | SRS10484393 | human | China:Shandong |
| 30 | KPX | PDT001199555.1 | GCA_021871395.1 | SAMN18928085 |  | human | China:jiangsu |
| 31 | ff60 | ASM981191v1 | GCA_009811915.1 | SAMN12618108 |  | human | China:Zhejiang |
| 32 | 537 |  |  | SAMN18145472 |  | human | China:Zhejiang |
| 33 | 2039D |  |  | SAMN10473951 | SRS4075676 | human | China:Fujian |
| 34 | 1566D | PDT000432720.1 |  | SAMN10473945 | SRS4075670 | human | China:Fujian |
| 35 | X_43 |  |  | SAMN21366036 | SRS10099906 | human | China:Hunan |
| 36 | X_11 |  |  | SAMN21366004 | SRS10099874 | human | China:Hunan |
| 37 | A128 | PDT001130527.1 | GCA_021906935.1 | SAMN21397448 | SRS10129119 | human | China:Jilin |
| 38 | bat16 | ASM2249287v1 | GCA_022492875.1 | SAMN26209580 |  | human | Italy:Bologna |
| 39 | 1804704 |  |  | SAMN16558222 | SRS7589589 | human | USA |
| 40 | KPN1916 | PDT001178223.1 | GCA_021897375.1 |  |  | human | Australia:Melbourne |
| 41 | PBIO1994 | PDT000929593.1 | GCA_022025555.1 |  |  | human | Germany |
| 42 | B 18564/18 | PDT001216093.1 | GCA_021907115.1 |  |  | human | India:New Delhi |
| 43 | THO 004 | ASM1513877v1 | GCA_015138775.1 |  |  | human | Japan:Tokyo |
| 44 | KPN9749 |  |  | SAMN15750999 | SRS7159695 | human | Netherlands:Leiden |
| 45 | 20P167W | ASM1794228v1 | GCA_017942285.1 | SAMN16226488 | SRS7397698 | human | Thailand:Bangkok |
| 46 | ARGID_33503 |  | GCA_922827125.1 | SAMEA11350784 | GCA_922827125.1 | human | United Kingdom |
| 47 | 5232 | ASM1939197v1 | GCA_019391975.1 | SAMN20338552 |  | human | Russia:Saint-Petersburg |
| 48 | 82A | ASM1211365v1 |  | SAMN15502221 |  | human | Pakistan |
| 49 | Bckp067 | ASM1216707v1 |  | SAMN14487965 |  | cow | China:Shandong |
| 50 | Bckp101 | ASM1216738v1 | GCA_012167075.1 | SAMN14487860 |  | cow | China:Shandong |
| 51 | Bckp206 | ASM1216721v1 |  | SAMN14487893 |  | cow | China:Heilongjiang |
| 52 | Bckp186 | ASM1216691v1 |  | SAMN14487873 |  | cow | China:Hebei |
| 53 | Bckp021 | ASM1211363v1 | GCA_012166915.1 | SAMN14487838 |  | cow | China:Jiangxi |
| 54 | Bckp212 | ASM1211361v1 | GCA_012113635.1 | SAMN14486704 |  | cow | China:Anhui |
| 55 | Bckp091 |  | GCA_012113615.1 | SAMN14486595 |  | cow | China:Jiangsu |
| 56 | hvK2115 |  | GCA_023347665.1 | SAMN25087048 |  | cow | China |
| 57 | HMKU VET |  | GCA_016888485.1 | SAMN17773724 |  | cow | Turkey:Hatay |
| 58 | c10 |  | GCA_021020755.1 | SAMN20525640 |  | cow | China |
| 59 | Ke33 |  | GCA_021020715.1 | SAMN20525726 |  | cow | China |
| 60 | C23 |  | GCA_002856075.1 | SAMN07793251 |  | cow | USA:New York |
| 61 | D36 |  | GCA_002856455.1 | SAMN07793273 |  | cow | USA:New York |
| 62 | EGKP5 |  | GCA_022341465.1 | SAMN14858806 |  | cattle | Egypt:Giza |
| 63 | EGKP6 |  | GCA_017161155.1 | SAMN14858807 |  | cattle | Egypt:Giza |
| 64 | NC 01 |  | GCA_019900305.2 | SAMN20362514 |  | cattle | USA |
| 65 | NY 06 | 17261_7#1 | GCA_019900805.2 | SAMN20362516 |  | cattle | USA |
| 66 | VRCO0033 |  | GCA_900171815.1 | SAMEA3472024 |  | cattle | United Kingdom |
| 67 | VRCO0034 |  | GCA_900172105.1 | SAMEA3472025 |  | cattle | United Kingdom |
| 68 | CVUAS 5901.5 |  | GCA_019678075.1 | SAMN07319195 |  | cattle | Germany |
| 69 | CVUAS 1678 |  | GCA_019426425.1 | SAMN07310305 |  | cattle | Germany |
| 70 | LZSYQHXN005 |  | GCA_025770285.1 | SAMN31205649 |  | cattle | China:Qinghai |
| 71 | 32 | ASM1452626v2 |  |  |  | chicken | China:Shandong |
| 72 | EGKP7 | ASM1716148v1 | GCA_017161485.1 |  |  | chicken | Egypt:Giza |
| 73 | Kp73 | ASM2451738v1 | GCA_024517385.1 |  |  | dog | China:Beijing |
| 74 | Kp59 | ASM2451744v1 | GCA_024517445.1 |  |  | cat | China:Beijing |
| 75 | 19KM1053 | ASM1341610v1 | GCA_013416105.1 |  |  | cat | Switzerland:Bern |
| 76 | Kp81 | ASM2451728v1 | GCA_024517285.1 |  |  | dog | China:Beijing |
| 77 | M T3 X3 | ASM1107716v1 | GCA_011077165.1 |  |  | swine | China:Shandong |
| 78 | K 1L | ASM2002346v1 | GCA_020023465.1 |  |  | swine | [China:Guangxi](https://www.ncbi.nlm.nih.gov/biosample?term=%22geo_loc_name=China:Guangxi%22%5battr%5d) |
| 79 | 194 | ASM432143v1 | GCA_004321435.1 |  |  | bird | China |
| 80 | QH276 1 1 | ASM432128v1 | GCA_004321285.1 |  |  | bird | China |
| 81 | GF24B | ASM412768v1 | GCA_004127685.1 |  |  | fly | China:Shandong |
| 82 | GF48B |  | GCA_004127855.1 |  |  | fly | China:Shandong |
| 83 | CVUAS 30299 | ASM1967918v1 | GCA_019679185.1 |  |  | horse | Germany |
| 84 | K | ASM799349v1 | GCA_007993495.1 |  |  | horse | Portugal:Lisbon |
| 85 | R50 | ASM697416v1 | GCA_006974165.1 |  |  | Rabbit | China:Zhejiang |
| 86 | CVUAS 7090.4 | ASM1967814v1 | GCA_019678145.1 |  |  | Rabbit | Germany |
| 87 | 015134X1B4 | ASM1838357v1 | GCA_018383575.1 |  |  | mouse | China:Sichuan |
| 88 | KPC | ASM1599927v1 | GCA_015999275.1 |  |  | mouse | USA:Cambridge, MA |
| 89 | KP20194c4 | ASM1334900v1 | GCA_013349005.1 |  |  | environment | China:Hunan |
| 90 | M 6 | ASM1107822v1 | GCA_011078225.1 |  |  | environment | China:Shandong |
| 91 | ZZW20 | ASM1342382v1 | GCA_013423825.1 |  |  | environment | China:Fujian |
| 92 | W2 5 ERG7 | 18090_8#79 | GCA_900093285.1 |  |  | environment | Thailand |
| 93 | KpN08 | ASM188438v1 | GCA_001884385.1 |  |  | environment | Canada:Calgary |
| 94 | SB611 | ASM2052598v1 | GCA_020525985.1 |  |  | environment | Netherlands |
| 95 | NCS43A | Q1947 | GCA_903166445.1 |  |  | environment | Marseille, France, Senegal |

**Supplementary Table 3** Bioinformatic analysis of 44 new Klebsiella pneumoniae isolates

| **Strain** | **ST** | **gapA** | **infB** | **mdh** | **pgi** | **phoE** | **rpoB** | tonB | **K type** | **wzi** | **K_locus** | **O_type** | **O LOCUS** | **AMR genes** | **Virulence genes** |
| --- | --- | --- | --- | --- | --- | --- | --- | --- | --- | --- | --- | --- | --- | --- | --- |
| kp102 | ST252 | 2 | 5 | 1 | 1 | 9 | 1 | 6 | K51 | wzi104 | KL51 | O1 | O1/O2v2 | blaSHV-1,fosA,oqxA,oqxB | acrA,acrB,clpV/tssH,cpsACP,dotU/tssL,entA,entB,entC,entE,entF,fepA,fepB,fepC,fepD,fepG,fes,fimA,fimB,fimC,fimD,fimE,fimF,fimG,fimH,fimI,fimK,galF,glf,gnd,hcp/tssD,icmF/tssM,iroE,kfoC,mrkA,mrkF,mrkH,mrkI,mrkJ,rcsA,rcsB,sciN/tssJ,tssF,tssG,ugd,vasE/tssK,vipA/tssB,vipB/tssC,wbbM,wbbN,wbbO,wzi,wzm,wzt,yagW/ecpD,yagX/ecpC,yagY/ecpB,yagZ/ecpA,ybdA,ykgK/ecpR |
| kp110 | ST3132 | 2 | 1 | 1 | 37 | 10 | 1 | 9 | unknown | wzi267 | KL142 | O1 | O1/O2v1 | blaSHV-27,fosA,oqxA,oqxB | acrA,acrB,clpV/tssH,cpsACP,dotU/tssL,entA,entB,entC,entE,entF,fepA,fepB,fepC,fepD,fepG,fes,fimA,fimB,fimC,fimD,fimE,fimF,fimG,fimH,fimI,fimK,galF,glf,gnd,hcp/tssD,icmF/tssM,iroE,mrkA,mrkB,mrkD,mrkH,mrkI,rcsA,rcsB,sciN/tssJ,tssF,tssG,ugd,vasE/tssK,vipA/tssB,vipB/tssC,wbbM,wbbN,wbbO,wzi,wzm,wzt,yagW/ecpD,yagX/ecpC,yagY/ecpB,yagZ/ecpA,ybdA,ykgK/ecpR |
| kp111 | ST76 | 4 | 1 | 1 | 1 | 21 | 1 | 35 | K10 | wzi100 | KL10 | O3/O3a | O3/O3a | blaSHV-1,fosA,oqxA,oqxB,tet(D) | acrA,acrB,clpV/tssH,cpsACP,dotU/tssL,entA,entB,entC,entE,entF,fepA,fepB,fepC,fepD,fepG,fes,fimA,fimB,fimC,fimD,fimE,fimF,fimG,fimH,fimI,fimK,galF,gnd,hcp/tssD,icmF/tssM,iroE,manC,mrkA,mrkB,mrkC,mrkD,mrkF,mrkH,mrkI,mrkJ,rcsA,rcsB,sciN/tssJ,tssF,tssG,ugd,vasE/tssK,vipA/tssB,vipB/tssC,wza,wzi,yagW/ecpD,yagX/ecpC,yagY/ecpB,yagZ/ecpA,ybdA,ykgK/ecpR |
| kp112 | ST76 | 4 | 1 | 1 | 1 | 21 | 1 | 35 | K10 | wzi100 | KL10 | O3/O3a | O3/O3a | fosA,oqxA,oqxB,tet(D) | acrA,acrB,clpV/tssH,cpsACP,dotU/tssL,entA,entB,entC,entE,entF,fepA,fepB,fepC,fepD,fepG,fes,fimA,fimB,fimC,fimD,fimE,fimF,fimG,fimH,fimI,fimK,galF,gnd,hcp/tssD,icmF/tssM,iroE,manC,mrkA,mrkB,mrkC,mrkD,mrkF,mrkH,mrkI,mrkJ,rcsA,rcsB,sciN/tssJ,tssF,tssG,ugd,vasE/tssK,vipA/tssB,vipB/tssC,wza,wzi,yagW/ecpD,yagX/ecpC,yagY/ecpB,yagZ/ecpA,ybdA,ykgK/ecpR |
| kp123 | ST76 | 4 | 1 | 1 | 1 | 21 | 1 | 35 | K10 | wzi100 | KL10 | O3/O3a | O3/O3a | fosA,oqxA,oqxB,tet(D) | acrA,acrB,clpV/tssH,cpsACP,dotU/tssL,entA,entB,entC,entE,entF,fepA,fepB,fepC,fepD,fepG,fes,fimA,fimB,fimC,fimD,fimE,fimF,fimG,fimH,fimI,fimK,galF,gnd,hcp/tssD,icmF/tssM,iroE,manC,mrkA,mrkB,mrkC,mrkD,mrkF,mrkH,mrkI,mrkJ,rcsA,rcsB,sciN/tssJ,tssF,tssG,ugd,vasE/tssK,vipA/tssB,vipB/tssC,wza,wzi,yagW/ecpD,yagX/ecpC,yagY/ecpB,yagZ/ecpA,ybdA,ykgK/ecpR |
| kp124 | ST76 | 4 | 1 | 1 | 1 | 21 | 1 | 35 | K10 | wzi100 | KL10 | O3/O3a | O3/O3a | blaSHV-1,fosA,oqxA,oqxB,tet(D) | acrA,acrB,clpV/tssH,cpsACP,dotU/tssL,entA,entB,entC,entE,entF,fepA,fepB,fepC,fepD,fepG,fes,fimA,fimB,fimC,fimD,fimE,fimF,fimG,fimH,fimI,fimK,galF,gnd,hcp/tssD,icmF/tssM,iroE,manC,mrkA,mrkB,mrkC,mrkD,mrkF,mrkH,mrkI,mrkJ,rcsA,rcsB,sciN/tssJ,tssF,tssG,ugd,vasE/tssK,vipA/tssB,vipB/tssC,wza,wzi,yagW/ecpD,yagX/ecpC,yagY/ecpB,yagZ/ecpA,ybdA,ykgK/ecpR |
| kp128 | ST76 | 4 | 1 | 1 | 1 | 21 | 1 | 35 | K10 | wzi100 | KL10 | O3/O3a | O3/O3a | blaSHV-1,fosA,oqxA,oqxB,tet(D) | acrA,acrB,clpV/tssH,cpsACP,dotU/tssL,entA,entB,entC,entE,entF,fepA,fepB,fepC,fepD,fepG,fes,fimA,fimB,fimC,fimD,fimE,fimF,fimG,fimH,fimI,fimK,galF,gnd,hcp/tssD,icmF/tssM,iroE,manC,mrkA,mrkB,mrkC,mrkD,mrkF,mrkH,mrkI,mrkJ,rcsA,rcsB,sciN/tssJ,tssF,tssG,ugd,vasE/tssK,vipA/tssB,vipB/tssC,wza,wzi,yagW/ecpD,yagX/ecpC,yagY/ecpB,yagZ/ecpA,ybdA,ykgK/ecpR |
| kp130 | ST76 | 4 | 1 | 1 | 1 | 21 | 1 | 35 | K10 | wzi100 | KL10 | O3/O3a | O3/O3a | blaSHV-1,fosA,oqxA,oqxB,tet(D) | acrA,acrB,clpV/tssH,cpsACP,dotU/tssL,entA,entB,entC,entE,entF,fepA,fepB,fepC,fepD,fepG,fes,fimA,fimB,fimC,fimD,fimE,fimF,fimG,fimH,fimI,fimK,galF,gnd,hcp/tssD,icmF/tssM,iroE,manC,mrkA,mrkB,mrkC,mrkD,mrkF,mrkH,mrkI,mrkJ,rcsA,rcsB,sciN/tssJ,tssF,tssG,ugd,vasE/tssK,vipA/tssB,vipB/tssC,wza,wzi,yagW/ecpD,yagX/ecpC,yagY/ecpB,yagZ/ecpA,ybdA,ykgK/ecpR |
| kp13BYS | ST1985 | 4 | 3 | 2 | 1 | 9 | 1 | 12 | K14 | wzi14 | KL14 | O3b | O3b | blaSHV-38,fosA5,oqxA,oqxB | KPHS_23120,acrA,acrB,clpV/tssH,cpsACP,dotU/tssL,entA,entB,entC,entE,entF,fepA,fepB,fepC,fepD,fepG,fes,fimA,fimB,fimC,fimD,fimE,fimF,fimG,fimH,fimI,fimK,galF,gnd,hcp/tssD,icmF/tssM,impA/tssA,iroE,manC,mrkA,mrkB,mrkC,mrkD,mrkF,mrkH,mrkI,mrkJ,rcsA,rcsB,sciN/tssJ,tli1,tssF,tssG,ugd,vasE/tssK,vgrG/tssI,vipA/tssB,vipB/tssC,wzi,yagW/ecpD,yagX/ecpC,yagY/ecpB,yagZ/ecpA,ybdA,ykgK/ecpR |
| kp16BYS | ST1985 | 4 | 3 | 2 | 1 | 9 | 1 | 12 | K14 | wzi14 | KL14 | O3b | O3b | blaSHV-38,fosA5,oqxA,oqxB | KPHS_23120,acrA,acrB,clpV/tssH,cpsACP,dotU/tssL,entA,entB,entC,entE,entF,fepA,fepB,fepC,fepD,fepG,fes,fimA,fimB,fimC,fimD,fimE,fimF,fimG,fimH,fimI,fimK,galF,gnd,hcp/tssD,icmF/tssM,impA/tssA,iroE,manC,mrkA,mrkB,mrkC,mrkD,mrkF,mrkH,mrkI,mrkJ,rcsA,rcsB,sciN/tssJ,tli1,tssF,tssG,ugd,vasE/tssK,vgrG/tssI,vipA/tssB,vipB/tssC,wzi,yagW/ecpD,yagX/ecpC,yagY/ecpB,yagZ/ecpA,ybdA,ykgK/ecpR |
| kp16GYS | ST12 | 6 | 3 | 1 | 1 | 12 | 1 | 4 | K35 | wzi162 | KL122 | O2afg | O1/O2v2 | blaSHV-107,fosA,oqxA,oqxB | KPHS_23120,acrA,acrB,clpV/tssH,cpsACP,dotU/tssL,entA,entB,entC,entE,entF,fepA,fepB,fepC,fepD,fepG,fes,fimA,fimB,fimC,fimD,fimE,fimF,fimG,fimH,fimI,fimK,galF,glf,gnd,hcp/tssD,icmF/tssM,impA/tssA,iroE,kfoC,manB,manC,mrkA,mrkB,mrkC,mrkD,mrkF,mrkH,mrkI,mrkJ,rcsA,rcsB,sciN/tssJ,tli1,tssF,tssG,vasE/tssK,vgrG/tssI,vipA/tssB,vipB/tssC,wbbM,wbbN,wbbO,wzi,wzm,wzt,yagW/ecpD,yagX/ecpC,yagY/ecpB,yagZ/ecpA,ybdA,ykgK/ecpR |
| kp17BYS | ST200 | 2 | 1 | 2 | 1 | 12 | 1 | 68 | K21 | wzi87 | KL21 | O3b | O3b | blaSHV-185,fosA,oqxA,oqxB | acrA,acrB,clpV/tssH,cpsACP,dotU/tssL,entA,entB,entC,entE,entF,fepA,fepB,fepC,fepD,fepG,fes,fimA,fimB,fimC,fimD,fimE,fimF,fimG,fimH,fimI,fimK,galF,gnd,hcp/tssD,icmF/tssM,iroE,manB,manC,mrkA,mrkB,mrkC,mrkD,mrkF,mrkH,mrkI,mrkJ,rcsA,rcsB,sciN/tssJ,tssF,tssG,ugd,vasE/tssK,vipA/tssB,vipB/tssC,wzi,yagW/ecpD,yagX/ecpC,yagY/ecpB,yagZ/ecpA,ybdA,ykgK/ecpR |
| kp1GYS | ST6290 | 51 | 1 | 5 | 1 | 9 | 357 | 13 | unknown | wzi442 | KL167 | OL101 | OL101 | blaSHV-40,fosA,oqxA,oqxB | acrA,acrB,entA,entB,entC,entD,entE,entF,fepA,fepB,fepC,fepD,fepG,fes,fimA,fimB,fimC,fimD,fimE,fimF,fimG,fimH,fimI,fimK,galF,gnd,iroE,mrkA,mrkB,mrkC,mrkD,mrkF,mrkH,mrkI,mrkJ,rcsA,rcsB,ugd,yagW/ecpD,yagX/ecpC,yagY/ecpB,yagZ/ecpA,ybdA,ykgK/ecpR |
| kp20BYS | ST2415 | 6 | 1 | 1 | 1 | 9 | 1 | 43 | unknown | wzi363 | KL131 | O4 | O4 | blaSHV-110,fosA,oqxA,oqxB | acrA,acrB,clpV/tssH,cpsACP,dotU/tssL,entA,entB,entC,entE,entF,fepA,fepB,fepC,fepD,fepG,fes,fimA,fimB,fimC,fimD,fimE,fimF,fimG,fimH,fimI,fimK,galF,gnd,hcp/tssD,icmF/tssM,iroE,mrkA,mrkB,mrkC,mrkD,mrkF,mrkH,mrkI,mrkJ,rcsA,rcsB,sciN/tssJ,tssF,tssG,ugd,vasE/tssK,vipA/tssB,vipB/tssC,wzi,yagW/ecpD,yagX/ecpC,yagY/ecpB,yagZ/ecpA,ybdA,ykgK/ecpR |
| kp2GYS | ST6290 | 51 | 1 | 5 | 1 | 9 | 357 | 13 | unknown | wzi442 | KL167 | OL101 | OL101 | blaSHV-40,fosA,oqxA,oqxB | acrA,acrB,entA,entB,entC,entD,entE,entF,fepA,fepB,fepC,fepD,fepG,fes,fimA,fimB,fimC,fimD,fimE,fimF,fimG,fimH,fimI,fimK,galF,gnd,iroE,mrkA,mrkB,mrkC,mrkD,mrkF,mrkH,mrkI,mrkJ,rcsA,rcsB,ugd,yagW/ecpD,yagX/ecpC,yagY/ecpB,yagZ/ecpA,ybdA,ykgK/ecpR |
| kp332 | ST3175 | 2 | 4 | 5 | 1 | 1 | 1 | 40 | unknown | wzi551 | KL171 | O3b | O3b | aph(3')-Ia,blaSHV-172,blaTEM-176,floR,fosA,oqxA,oqxB,qnrS1,tet(A) | acrA,acrB,allA,allB,allC,allD,allR,allS,clpV/tssH,cpsACP,dotU/tssL,entA,entB,entC,entE,entF,fepA,fepB,fepC,fepD,fepG,fes,fimA,fimB,fimC,fimD,fimE,fimF,fimG,fimH,fimI,fimK,galF,gnd,hcp/tssD,icmF/tssM,impA/tssA,iroE,manB,manC,mrkA,mrkB,mrkC,mrkD,mrkF,mrkH,mrkI,mrkJ,rcsA,rcsB,sciN/tssJ,tssF,tssG,vasE/tssK,vipA/tssB,vipB/tssC,wza,wzi,yagW/ecpD,yagX/ecpC,yagY/ecpB,yagZ/ecpA,ybdA,ykgK/ecpR |
| kp335 | ST200 | 2 | 1 | 2 | 1 | 12 | 1 | 68 | unknown | wzi493 | KL139 | O3b | O3b | blaSHV-185,fosA,oqxA,oqxB | acrA,acrB,clpV/tssH,cpsACP,dotU/tssL,entA,entB,entC,entE,entF,fepA,fepB,fepC,fepD,fepG,fes,fimA,fimB,fimC,fimD,fimE,fimF,fimG,fimH,fimI,fimK,galF,gnd,hcp/tssD,icmF/tssM,iroE,manB,manC,mrkA,mrkB,mrkC,mrkD,mrkF,mrkH,mrkI,mrkJ,rcsA,rcsB,sciN/tssJ,tssF,tssG,ugd,vasE/tssK,vipA/tssB,vipB/tssC,wzi,yagW/ecpD,yagX/ecpC,yagY/ecpB,yagZ/ecpA,ybdA,ykgK/ecpR |
| kp43BYS | ST857 | 2 | 35 | 2 | 35 | 56 | 24 | 19 | unknown | wzi181 | KL133 | O2a | O1/O2v1 | blaSHV-33,fosA,oqxA,oqxB | acrA,acrB,clpV/tssH,cpsACP,dotU/tssL,entA,entB,entC,entE,entF,fepA,fepB,fepC,fepD,fepG,fes,fimA,fimB,fimC,fimD,fimE,fimF,fimG,fimH,fimI,fimK,galF,glf,gnd,hcp/tssD,icmF/tssM,impA/tssA,iroE,mrkA,mrkB,mrkC,mrkD,mrkF,mrkH,mrkI,mrkJ,rcsA,rcsB,sciN/tssJ,tssF,tssG,ugd,vasE/tssK,vipA/tssB,vipB/tssC,wbbM,wbbN,wbbO,wzm,wzt,yagW/ecpD,yagX/ecpC,yagY/ecpB,yagZ/ecpA,ybdA,ykgK/ecpR |
| kp593 | ST6317 | 3 | 16 | 1 | 1 | 20 | 1 | 40 | K18 | wzi738 | KL18 | O4 | O4 | blaSHV-40,fosA,oqxA,oqxB | KPHS_23120,acrA,acrB,clpV/tssH,cpsACP,dotU/tssL,entA,entB,entC,entE,entF,fepA,fepB,fepC,fepD,fepG,fes,fimA,fimB,fimC,fimD,fimE,fimF,fimG,fimH,fimI,fimK,galF,gnd,hcp/tssD,icmF/tssM,impA/tssA,iroE,mrkA,mrkB,mrkC,mrkD,mrkF,mrkH,mrkI,mrkJ,rcsA,rcsB,sciN/tssJ,tli1,tssF,tssG,ugd,vasE/tssK,vgrG/tssI,vipA/tssB,vipB/tssC,wza,wzi,yagW/ecpD,yagX/ecpC,yagY/ecpB,yagZ/ecpA,ybdA,ykgK/ecpR |
| kp5BYS | ST857 | 2 | 35 | 2 | 35 | 56 | 24 | 19 | unknown | wzi181 | KL133 | O2a | O1/O2v1 | blaSHV-33,fosA,oqxA,oqxB | acrA,acrB,clpV/tssH,cpsACP,dotU/tssL,entA,entB,entC,entE,entF,fepA,fepB,fepC,fepD,fepG,fes,fimA,fimB,fimC,fimD,fimE,fimF,fimG,fimH,fimI,fimK,galF,glf,gnd,hcp/tssD,icmF/tssM,impA/tssA,iroE,mrkA,mrkB,mrkC,mrkD,mrkF,mrkH,mrkI,mrkJ,rcsA,rcsB,sciN/tssJ,tssF,tssG,ugd,vasE/tssK,vipA/tssB,vipB/tssC,wbbM,wbbN,wbbO,wzm,wzt,yagW/ecpD,yagX/ecpC,yagY/ecpB,yagZ/ecpA,ybdA,ykgK/ecpR |
| kp5GYS | ST6290 | 51 | 1 | 5 | 1 | 9 | 357 | 13 | unknown | wzi442 | KL167 | OL101 | OL101 | blaSHV-40,fosA,oqxA,oqxB | acrA,acrB,entA,entB,entC,entD,entE,entF,fepA,fepB,fepC,fepD,fepG,fes,fimA,fimB,fimC,fimD,fimE,fimF,fimG,fimH,fimI,fimK,galF,gnd,iroE,mrkA,mrkB,mrkC,mrkD,mrkF,mrkH,mrkI,mrkJ,rcsA,rcsB,ugd,yagW/ecpD,yagX/ecpC,yagY/ecpB,yagZ/ecpA,ybdA,ykgK/ecpR |
| kp603 | ST2406 | 3 | 106 | 20 | 18 | 271 | 4 | 4 | K6 | wzi316 | KL6 | O2a | O1/O2v1 | blaSHV-185,fosA5,oqxA,oqxB | KPHS_23120,acrA,acrB,clpV/tssH,cpsACP,dotU/tssL,entA,entB,entC,entE,entF,fepA,fepB,fepC,fepD,fepG,fes,fimA,fimB,fimC,fimD,fimE,fimF,fimG,fimH,fimI,fimK,galF,glf,gnd,hcp/tssD,icmF/tssM,impA/tssA,iroE,mrkA,mrkB,mrkC,mrkD,mrkF,mrkH,mrkI,mrkJ,rcsA,rcsB,sciN/tssJ,tli1,tssF,tssG,ugd,vasE/tssK,vgrG/tssI,vipA/tssB,vipB/tssC,wbbM,wbbN,wbbO,wzi,wzm,wzt,yagW/ecpD,yagX/ecpC,yagY/ecpB,yagZ/ecpA,ybdA,ykgK/ecpR |
| kp617 | ST4428 | 2 | 5 | 2 | 6 | 3 | 1 | 4 | unknown | wzi643 | KL173 | O3b | O3b | blaSHV-148,fosA,oqxA,oqxB | acrA,acrB,clpV/tssH,cpsACP,dotU/tssL,entA,entB,entC,entE,entF,fepA,fepB,fepC,fepD,fepG,fes,fimA,fimB,fimC,fimD,fimE,fimF,fimG,fimH,fimI,fimK,galF,gnd,hcp/tssD,icmF/tssM,iroE,manB,mrkA,mrkB,mrkC,mrkD,mrkF,mrkH,mrkI,mrkJ,rcsA,rcsB,sciN/tssJ,tssF,tssG,ugd,vasE/tssK,vipA/tssB,vipB/tssC,wzi,yagW/ecpD,yagX/ecpC,yagY/ecpB,yagZ/ecpA,ybdA,ykgK/ecpR |
| kp622 | ST4642 | 2 | 31 | 2 | 1 | 9 | 4 | 23 | K48 | wzi737 | KL48 | O2a | O1/O2v1 | blaSHV-40,fosA,oqxA,oqxB | acrA,acrB,allA,allB,allC,allD,allR,allS,astA,clpV/tssH,cpsACP,dotU/tssL,entA,entB,entC,entD,entE,entF,fepA,fepB,fepC,fepD,fepG,fes,fimA,fimB,fimC,fimD,fimE,fimF,fimG,fimH,fimI,fimK,galF,glf,gnd,hcp/tssD,icmF/tssM,iroE,mrkA,mrkB,mrkC,mrkD,mrkF,mrkH,mrkI,mrkJ,rcsA,rcsB,sciN/tssJ,tssF,tssG,ugd,vasE/tssK,vipA/tssB,vipB/tssC,wbbM,wbbN,wbbO,wzi,wzm,wzt,yagW/ecpD,yagX/ecpC,yagY/ecpB,yagZ/ecpA,ybdA,ykgK/ecpR |
| kp628 | ST4428 | 2 | 5 | 2 | 6 | 3 | 1 | 4 | unknown | wzi643 | KL173 | O3b | O3b | blaSHV-148,fosA,oqxA,oqxB | acrA,acrB,clpV/tssH,cpsACP,dotU/tssL,entA,entB,entC,entE,entF,fepA,fepB,fepC,fepD,fepG,fes,fimA,fimB,fimC,fimD,fimE,fimF,fimG,fimH,fimI,fimK,galF,gnd,hcp/tssD,icmF/tssM,iroE,manB,mrkA,mrkB,mrkC,mrkD,mrkF,mrkH,mrkI,mrkJ,rcsA,rcsB,sciN/tssJ,tssF,tssG,ugd,vasE/tssK,vipA/tssB,vipB/tssC,wzi,yagW/ecpD,yagX/ecpC,yagY/ecpB,yagZ/ecpA,ybdA,ykgK/ecpR |
| kp632 | ST37 | 2 | 9 | 2 | 1 | 13 | 1 | 16 | K38 | wzi96 | KL38 | O3b | O3b | aph(3'')-Ib,aph(6)-Id,blaSHV-110,fosA,oqxA,oqxB,tet(B) | acrA,acrB,clpV/tssH,cpsACP,dotU/tssL,entA,entB,entC,entD,entE,entF,fepA,fepB,fepC,fepD,fepG,fes,fimA,fimB,fimC,fimD,fimE,fimF,fimG,fimH,fimI,fimK,galF,gnd,hcp/tssD,icmF/tssM,iroE,manB,manC,mrkA,mrkB,mrkC,mrkD,mrkF,mrkH,mrkI,mrkJ,rcsA,rcsB,sciN/tssJ,tssF,tssG,ugd,vasE/tssK,vipA/tssB,vipB/tssC,wzi,yagW/ecpD,yagX/ecpC,yagY/ecpB,yagZ/ecpA,ybdA,ykgK/ecpR |
| kp647 | ST147 | 3 | 4 | 6 | 1 | 7 | 4 | 38 | K81 | wzi81 | KL81 | OL101 | OL101 | ARR-3,aac(3)-IId,aac(6')-Ib-cr,aadA16,aph(3'')-Ib,aph(3')-Ia,aph(6)-Id,blaLAP-2,dfrA27,floR,fosA,mph(A),oqxA,oqxB,qnrS1,sul1,sul2,tet(A) | KPHS_23120,acrA,acrB,clpV/tssH,cpsACP,dotU/tssL,entA,entB,entC,entE,entF,fepA,fepB,fepC,fepD,fepG,fes,fimA,fimB,fimC,fimD,fimE,fimF,fimG,fimH,fimI,fimK,galF,gnd,hcp/tssD,icmF/tssM,impA/tssA,iroE,mrkA,mrkB,mrkC,mrkD,mrkF,mrkH,mrkI,mrkJ,rcsA,rcsB,sciN/tssJ,tli1,tssF,tssG,ugd,vasE/tssK,vgrG/tssI,vipA/tssB,vipB/tssC,wzi,yagW/ecpD,yagX/ecpC,yagY/ecpB,yagZ/ecpA,ybdA,ykgK/ecpR |
| kp681 | ST294 | 2 | 1 | 2 | 1 | 4 | 4 | 87 | K1 | wzi128 | KL1 | O1 | O1/O2v2 | blaSHV-187,fosA,oqxA,oqxB | acrA,acrB,clpV/tssH,cpsACP,dotU/tssL,entA,entB,entC,entE,entF,fepA,fepB,fepC,fepD,fepG,fes,fimA,fimB,fimC,fimD,fimE,fimF,fimG,fimH,fimI,fimK,galF,glf,gmd,gnd,hcp/tssD,icmF/tssM,impA/tssA,iroE,kfoC,magA/wzy_K1,manB,manC,mrkA,mrkB,mrkC,mrkD,mrkF,mrkH,mrkI,mrkJ,rcsA,rcsB,sciN/tssJ,tssF,tssG,ugd,vasE/tssK,vipA/tssB,vipB/tssC,wbbM,wbbN,wbbO,wcaH,wcaI,wcaJ,wclY,wcsS,wcsT,wza,wzb,wzc,wzi,wzm,wzt,wzx,yagW/ecpD,yagX/ecpC,yagY/ecpB,yagZ/ecpA,ybdA,ykgK/ecpR |
| kp684 | ST605 | 4 | 35 | 2 | 1 | 112 | 7 | 70 | K58 | wzi130 | KL58 | O3b | O3b | blaSHV-144,fosA,oqxA,oqxB | acrA,acrB,allA,allB,allC,allD,allR,allS,clpV/tssH,cpsACP,dotU/tssL,entA,entB,entC,entE,entF,fepA,fepB,fepC,fepD,fepG,fes,fimA,fimB,fimC,fimD,fimE,fimF,fimG,fimH,fimI,fimK,galF,gmd,gnd,hcp/tssD,icmF/tssM,iroE,manC,mrkA,mrkB,mrkC,mrkD,mrkF,mrkH,mrkI,mrkJ,rcsA,rcsB,sciN/tssJ,tssF,tssG,ugd,vasE/tssK,vipA/tssB,vipB/tssC,wcaH,wcaI,wcaJ,wzi,yagW/ecpD,yagX/ecpC,yagY/ecpB,yagZ/ecpA,ybdA,ykgK/ecpR |
| kp6BYS | ST6290 | 51 | 1 | 5 | 1 | 9 | 357 | 13 | unknown | wzi442 | KL167 | OL101 | OL101 | blaSHV-40,fosA,oqxA,oqxB | acrA,acrB,entA,entB,entC,entD,entE,entF,fepA,fepB,fepC,fepD,fepG,fes,fimA,fimB,fimC,fimD,fimE,fimF,fimG,fimH,fimI,fimK,galF,gnd,iroE,mrkA,mrkB,mrkC,mrkD,mrkF,mrkH,mrkI,mrkJ,rcsA,rcsB,ugd,yagW/ecpD,yagX/ecpC,yagY/ecpB,yagZ/ecpA,ybdA,ykgK/ecpR |
| kp6GYS | ST6290 | 51 | 1 | 5 | 1 | 9 | 357 | 13 | unknown | wzi442 | KL167 | OL101 | OL101 | blaSHV-40,fosA,oqxA,oqxB | acrA,acrB,entA,entB,entC,entD,entE,entF,fepA,fepB,fepC,fepD,fepG,fes,fimA,fimB,fimC,fimD,fimE,fimF,fimG,fimH,fimI,fimK,galF,gnd,iroE,mrkA,mrkB,mrkC,mrkD,mrkF,mrkH,mrkI,mrkJ,rcsA,rcsB,ugd,yagW/ecpD,yagX/ecpC,yagY/ecpB,yagZ/ecpA,ybdA,ykgK/ecpR |
| kp710 | ST4251 | 2 | 1 | 2 | 1 | 12 | 1 | 136 | K31 | wzi102 | KL31 | O3/O3a | O3/O3a | blaSHV-11,fosA,oqxA,oqxB | acrA,acrB,clpV/tssH,cpsACP,dotU/tssL,entA,entB,entC,entE,entF,fepA,fepB,fepC,fepD,fepG,fes,fimA,fimB,fimC,fimD,fimE,fimF,fimG,fimH,fimI,fimK,galF,gnd,hcp/tssD,icmF/tssM,iroE,manB,mrkA,mrkB,mrkC,mrkD,mrkF,mrkH,mrkI,mrkJ,rcsA,rcsB,sciN/tssJ,tssF,tssG,ugd,vasE/tssK,vipA/tssB,vipB/tssC,wzi,yagW/ecpD,yagX/ecpC,yagY/ecpB,yagZ/ecpA,ybdA,ykgK/ecpR |
| kp711 | ST37 | 2 | 9 | 2 | 1 | 13 | 1 | 16 | K12 | wzi12 | KL12 | OL103 | OL103 | blaSHV-110,fosA,oqxA,oqxB | acrA,acrB,clpV/tssH,cpsACP,dotU/tssL,entA,entB,entC,entD,entE,entF,fepA,fepB,fepC,fepD,fepG,fes,fimA,fimB,fimC,fimD,fimE,fimF,fimG,fimH,fimI,fimK,galF,gnd,hcp/tssD,icmF/tssM,iroE,mrkA,mrkB,mrkC,mrkD,mrkF,mrkH,mrkI,mrkJ,rcsA,rcsB,sciN/tssJ,tssF,tssG,ugd,vasE/tssK,vipA/tssB,vipB/tssC,wzi,yagW/ecpD,yagX/ecpC,yagY/ecpB,yagZ/ecpA,ybdA,ykgK/ecpR |
| kp93 | ST252 | 2 | 5 | 1 | 1 | 9 | 1 | 6 | K51 | wzi104 | KL51 | O1 | O1/O2v2 | blaSHV-1,fosA,oqxA,oqxB | acrA,acrB,clpV/tssH,cpsACP,dotU/tssL,entA,entB,entC,entE,entF,fepA,fepB,fepC,fepD,fepG,fes,fimA,fimB,fimC,fimD,fimE,fimF,fimG,fimH,fimI,fimK,galF,glf,gnd,hcp/tssD,icmF/tssM,iroE,kfoC,mrkB,mrkC,mrkD,mrkF,mrkH,mrkI,mrkJ,rcsA,rcsB,sciN/tssJ,tssF,tssG,ugd,vasE/tssK,vipA/tssB,vipB/tssC,wbbM,wbbN,wbbO,wzi,wzm,wzt,yagW/ecpD,yagX/ecpC,yagY/ecpB,yagZ/ecpA,ybdA,ykgK/ecpR |
| kpL15N | ST29 | 2 | 3 | 2 | 2 | 6 | 4 | 4 | K54 | wzi115 | KL54 | O1 | O1/O2v2 | blaSHV-187,fosA,oqxA,oqxB | acrA,acrB,clpV/tssH,cpsACP,dotU/tssL,entA,entB,entC,entE,entF,fepA,fepB,fepC,fepD,fepG,fes,fimA,fimB,fimC,fimD,fimE,fimF,fimG,fimH,fimI,fimK,galF,glf,gnd,hcp/tssD,icmF/tssM,impA/tssA,iroE,kfoC,manB,manC,mrkA,mrkB,mrkC,mrkD,mrkF,mrkH,mrkI,mrkJ,rcsA,rcsB,sciN/tssJ,tssF,tssG,ugd,vasE/tssK,vipA/tssB,vipB/tssC,wbbM,wbbN,wbbO,wcaG,wcaH,wcaI,wcaJ,wzi,wzm,wzt,yagW/ecpD,yagX/ecpC,yagY/ecpB,yagZ/ecpA,ybdA,ykgK/ecpR |
| kpY40B | ST857 | 2 | 35 | 2 | 35 | 56 | 24 | 19 | unknown | wzi181 | KL133 | O2a | O1/O2v1 | blaSHV-33,fosA,oqxA,oqxB | acrA,acrB,clpV/tssH,cpsACP,dotU/tssL,entA,entB,entC,entE,entF,fepA,fepB,fepC,fepD,fepG,fes,fimA,fimB,fimC,fimD,fimE,fimF,fimG,fimH,fimI,fimK,galF,glf,gnd,hcp/tssD,icmF/tssM,impA/tssA,iroE,mrkA,mrkB,mrkC,mrkD,mrkF,mrkH,mrkI,mrkJ,rcsA,rcsB,sciN/tssJ,tssF,tssG,ugd,vasE/tssK,vipA/tssB,vipB/tssC,wbbM,wbbN,wbbO,wzm,wzt,yagW/ecpD,yagX/ecpC,yagY/ecpB,yagZ/ecpA,ybdA,ykgK/ecpR |
| kpY41GYS | ST611 | 5 | 1 | 5 | 1 | 7 | 11 | 24 | unknown | wzi442 | KL167 | OL101 | OL101 | blaSHV-27,fosA,oqxA,oqxB | acrA,acrB,clpV/tssH,dotU/tssL,entA,entB,entC,entE,entF,fepA,fepB,fepC,fepD,fepG,fes,fimA,fimB,fimC,fimD,fimE,fimF,fimG,fimH,fimI,fimK,galF,gnd,hcp/tssD,icmF/tssM,impA/tssA,iroE,mrkA,mrkB,mrkC,mrkD,mrkF,mrkH,mrkI,mrkJ,rcsA,rcsB,sciN/tssJ,tssF,tssG,ugd,vasE/tssK,vipA/tssB,yagW/ecpD,yagX/ecpC,yagY/ecpB,yagZ/ecpA,ybdA,ykgK/ecpR |
| kpY43B | ST857 | 2 | 35 | 2 | 35 | 56 | 24 | 19 | unknown | wzi181 | KL133 | O2a | O1/O2v1 | blaSHV-33,fosA,oqxA,oqxB | acrA,acrB,clpV/tssH,cpsACP,dotU/tssL,entA,entB,entC,entE,entF,fepA,fepB,fepC,fepD,fepG,fes,fimA,fimB,fimC,fimD,fimE,fimF,fimG,fimH,fimI,fimK,galF,glf,gnd,hcp/tssD,icmF/tssM,impA/tssA,iroE,mrkA,mrkB,mrkC,mrkD,mrkF,mrkH,mrkI,mrkJ,rcsA,rcsB,sciN/tssJ,tssF,tssG,ugd,vasE/tssK,vipA/tssB,vipB/tssC,wbbM,wbbN,wbbO,wzm,wzt,yagW/ecpD,yagX/ecpC,yagY/ecpB,yagZ/ecpA,ybdA,ykgK/ecpR |
| kpYF3YS | ST6291 | 2 | 1 | 5 | 1 | 604 | 8 | 13 | K39 | wzi39 | KL39 | O3b | O3b | blaSHV-148,fosA,oqxA,oqxB | acrA,acrB,clpV/tssH,cpsACP,dotU/tssL,entA,entB,entC,entE,entF,fepA,fepB,fepC,fepD,fepG,fes,fimA,fimB,fimC,fimD,fimE,fimF,fimG,fimH,fimI,fimK,galF,gnd,hcp/tssD,icmF/tssM,impA/tssA,iroE,mrkA,mrkB,mrkC,mrkD,mrkF,mrkH,mrkI,mrkJ,rcsA,rcsB,sciN/tssJ,tssF,tssG,ugd,vasE/tssK,vipB/tssC,wzi,yagW/ecpD,yagX/ecpC,yagY/ecpB,yagZ/ecpA,ybdA,ykgK/ecpR |
| kpA667g5f | ST4428 | 2 | 5 | 2 | 6 | 3 | 1 | 4 | unknown | wzi643 | / | O3b | O3b | blaSHV-148,fosA,oqxA,oqxB | acrA,acrB,clpV/tssH,cpsACP,dotU/tssL,entA,entB,entC,entE,entF,fepA,fepB,fepC,fepD,fepG,fes,fimA,fimB,fimC,fimD,fimE,fimF,fimG,fimH,fimI,fimK,galF,gnd,hcp/tssD,icmF/tssM,iroE,manB,mrkA,mrkB,mrkC,mrkD,mrkF,mrkH,mrkI,mrkJ,rcsA,rcsB,sciN/tssJ,tssF,tssG,ugd,vasE/tssK,vipA/tssB,vipB/tssC,wzi,yagW/ecpD,yagX/ecpC,yagY/ecpB,yagZ/ecpA,ybdA,ykgK/ecpR |
| kpMH01 | ST2800 | 2 | 1 | 2 | 1 | 9 | 1 | 18 | unknown | wzi194 | KL108 | O1 | O1/O2v2 | aph(3'')-Ib,aph(6)-Id,blaSHV-82,fosA,oqxA,oqxB,tet(B) | acrA,acrB,clpV/tssH,cpsACP,dotU/tssL,entA,entB,entC,entE,entF,fepA,fepB,fepC,fepD,fepG,fes,fimA,fimB,fimC,fimD,fimE,fimF,fimG,fimH,fimI,fimK,galF,glf,gnd,hcp/tssD,icmF/tssM,impA/tssA,iroE,kfoC,manB,manC,mrkA,mrkB,mrkC,mrkD,mrkF,mrkH,mrkI,mrkJ,rcsA,rcsB,sciN/tssJ,tssF,tssG,ugd,vasE/tssK,vipA/tssB,vipB/tssC,wbbM,wbbN,wbbO,wzi,wzm,wzt,yagW/ecpD,yagX/ecpC,yagY/ecpB,yagZ/ecpA,ybdA,ykgK/ecpR |
| kpXJ | ST3040 | 2 | 1 | 1 | 1 | 7 | 4 | 61 | K38 | wzi281 | KL38 | O3b | O3b | aph(3'')-Ib,aph(6)-Id,blaSHV-82,fosA,oqxA,oqxB | KPHS_23120,acrA,acrB,clpV/tssH,cpsACP,dotU/tssL,entA,entB,entC,entE,entF,fepA,fepB,fepC,fepD,fepG,fes,fimA,fimB,fimC,fimD,fimE,fimF,fimG,fimH,fimI,fimK,galF,gnd,hcp/tssD,icmF/tssM,impA/tssA,iroE,manB,manC,mrkA,mrkB,mrkC,mrkD,mrkF,mrkH,mrkI,mrkJ,rcsA,rcsB,sciN/tssJ,tssF,tssG,ugd,vasE/tssK,vgrG/tssI,vipA/tssB,vipB/tssC,wzi,yagW/ecpD,yagX/ecpC,yagY/ecpB,yagZ/ecpA,ybdA,ykgK/ecpR |
| kpA710m5p | ST4251 | 2 | 1 | 2 | 1 | 12 | 1 | 136 | K31 | wzi102 | KL31 | O3/O3a | O3/O3a | blaSHV-11,fosA,oqxA,oqxB | acrA,acrB,clpV/tssH,cpsACP,dotU/tssL,entA,entB,entC,entE,entF,fepA,fepB,fepC,fepD,fepG,fes,fimA,fimB,fimC,fimD,fimE,fimF,fimG,fimH,fimI,fimK,galF,gnd,hcp/tssD,icmF/tssM,iroE,manB,manC,mrkA,mrkB,mrkC,mrkD,mrkF,mrkH,mrkI,mrkJ,rcsA,rcsB,sciN/tssJ,tssF,tssG,ugd,vasE/tssK,vipA/tssB,vipB/tssC,wzi,yagW/ecpD,yagX/ecpC,yagY/ecpB,yagZ/ecpA,ybdA,ykgK/ecpR |
| kpMT01 | ST234 | 2 | 1 | 2 | 1 | 7 | 1 | 24 | K35 | wzi163 | KL122 | O1 | O1/O2v2 | blaCTX-M-14,blaDHA-1,blaSHV-27,fosA,oqxA,oqxB,qnrB4,sul1 | acrA,acrB,clpV/tssH,cpsACP,dotU/tssL,entA,entB,entC,entE,entF,fepA,fepB,fepC,fepD,fepG,fes,fimA,fimB,fimC,fimD,fimE,fimF,fimG,fimH,fimI,fimK,galF,glf,gnd,hcp/tssD,icmF/tssM,iroE,kfoC,manB,manC,mrkA,mrkB,mrkC,mrkD,mrkF,mrkH,mrkI,mrkJ,rcsA,rcsB,sciN/tssJ,tssF,tssG,vasE/tssK,vipA/tssB,vipB/tssC,wbbM,wbbN,wbbO,wzi,wzm,wzt,yagW/ecpD,yagX/ecpC,yagY/ecpB,yagZ/ecpA,ybdA,ykgK/ecpR |
